# Passive wheels – A new localization system for automated guided vehicles

**DOI:** 10.1016/j.heliyon.2024.e34967

**Published:** 2024-07-20

**Authors:** Kacper Bereszyński, Marcin Pelic, Wojciech Paszkowiak, Stanisław Pabiszczak, Adam Myszkowski, Krzysztof Walas, Grzegorz Czechmanowski, Jan Węgrzynowski, Tomasz Bartkowiak

**Affiliations:** a Institute of Mechanical Technology, Poznan University of Technology, Poland; b Institute of Robotics and Machine Intelligence, Poznan University of Technology, Poland

**Keywords:** AGV, Mobile robots, Localization, Dead reckoning

## Abstract

This article introduces passive wheels as the new localization system for automated guided vehicles (AGVs). The article focuses on investigating the accuracy of the proposed system and comparing with other widely used solutions: rotary encoders coupled with drive wheels, AHRS and LiDAR scanner. The fusion of dead reckoning and inertial data is acquired by the implementation of the Kalman filter. On the other hand, the fusion of LiDAR data depends on application of the AMCL or the graph-based algorithm. The study was conducted on five different scenarios, designed to investigate the influence of specific types of movements on the performance of tested localization methods. Results indicate, that passive wheels dead reckoning outperforms drive wheels dead reckoning in most scenarios, minimizing errors due to reduced both longitudal and lateral slippages, which appear during AGV accelerating, decelerating and turning. AHRS integration improves accuracy, especially in scenarios involving significant amount of angular motion. LiDAR-based methods in short term show mediocre results, due to relatively high, but steady values of error. The study highlights the importance of the map's quality for LiDAR-based techniques and points up the conditions, under which the LiDAR-based techniques do not operate very well. In conclusion, the research provides insights into the strengths and weaknesses of various AGV localization techniques in various movement scenarios, emphasizing the impact of sensor choice and path-shape conditions on accuracy.

## Highlights

• New localization method based on passive wheels is introduced and compared with others.
• Passive wheels dead reckoning consistently outperforms drive wheels dead reckoning, reducing slippages.
• Fused encoders and IMU data markedly enhance accuracy in dead reckoning systems.
• Five different test scenarios allow a comprehensive framework for evaluating localization systems.
• Recommendations: Use passive wheels for short-term, fused LiDAR for sustained accuracy.

## 1 Introduction

When considering the design of an AGV (Automated Guided Vehicle) navigation system, several cooperating subsystems can be distinguished, each of which is responsible for performing a specific group of tasks. One such crucial subsystem is the localization system, tasked with furnishing precise information regarding the vehicle's position and orientation, with or without reference to objects located in workspace. In the past, before the widespread adoption of the technology based on virtual localization, most common methods involved the use of high-contrast lines painted on the floor and induction or magnetic lines placed under the floor to unambiguously determine the route followed by the vehicle [1].

The popularity of those methods can be explained by their simplicity and low cost of implementation. Yet, they come with certain drawbacks. Their most significant limitation is the inherent inability to effectively navigate around obstacles that may obstruct the vehicle's designated route. This, in turn, leads to the limited flexibility and reduced number of possible applications. To address this problem, new technologies have been developed using various types of proprioceptive and exteroceptive sensors [[2], [3], [4], [5], [6], [7]].

Those technologies include techniques based on LiDAR (Light Detection And Ranging) scanners [[8], [9], [10], [11], [12], [13], [14]], stereoscopic cameras, depth-sensing cameras [[15], [16], [17], [18], [19], [20]], IMU (Inertial Measurement Unit) [[21], [22], [23]], RFID (Radio Frequency Identification) sensors, ultrasonic sensors, or encoders mounted on the wheels or motors of the vehicle. There are also methods in which the distance from the vehicle to transceivers connected in a Wi-Fi or BLE (Bluetooth Low Energy) network is estimated by reading the RSSI (Received Signal Strength Indication) [[24], [25], [26], [27]]. The final localization can be calculated using triangulation or trilateration.

Laser localization of autonomous vehicles is mainly based on two methods. The first involves placing permanently fixed unique markers (also called landmarks) in the vehicle's workspace. The distance between the markers is known. The vehicle is equipped with a laser sensor, responsible for detecting the markers and feeding the control system with information about its distance from the currently detected markers. Then, basing on the readings and using the triangulation or trilateration method, the absolute position and orientation of the vehicle are determined [28,29].

The second localization method is natural localization using a LiDAR scanner. It involves matching the contours of the space currently being recorded by the scanner with the contours recorded on a previously generated two-dimensional map of the working area. This map is most often generated using SLAM (Simultaneous Localization And Mapping) algorithm. A method similar to natural localization with a LIDAR scanner involves the data from a stereoscopic or depth-sensing camera. Those devices capture an images which are converted into a three-dimensional point clouds in order to create a 3D map. The map serves as an absolute reference point for the vehicle localization algorithm [[30], [31], [32]].

The aforementioned methods operate on the principle of localizing a vehicle in relation to external elements such as maps or landmarks. Alternatively, there are techniques called dead reckoning, that calculate the current localization with reference to the initial position and orientation. The most common approach is to use rotary encoders that provide information about the change in the angular position of the vehicle's drive wheels, which is used to calculate wheels speed. By understanding the vehicle's kinematics, these speeds are then used to estimate both linear and angular velocities. Apart from rotary encoders, IMU can also be used to determine the velocity and acceleration of the vehicle or AHRS (Attitude and Heading Reference System) to obtain the absolute orientation of the vehicle.

In practice, the use of dead reckoning-based techniques leads to low-accuracy localization due to lateral and longitudinal slippage of wheels, which affect the quality of rotary encoder measurements, and signal noise associated with the use of inertial sensors. The wheels slippages (longitudinal and lateral) have the most influence during AGV accelerating, decelerating and turning. To mitigate the influence of those factors and enhance the efficacy of methods relying on absolute references, various sensor fusion approaches have been developed [[33], [34], [35], [36], [37], [38], [39], [40], [41], [42], [43], [44]]. Other approach was presented by Conner et al. [45]. In their work authors added additional sensing on the rear castor wheel differentially steered three-wheeled mobile robot, what improved the position estimation. However, that method presents some limitation for reversing due to the uncontrolled motion of the castor wheel.

The prevalent algorithms employed for sensor fusion encompass various adaptations of the Kalman filter, with the widely favored options being the Extended Kalman Filter (EKF) and the Unscented Kalman Filter (UKF). In scenarios where LiDAR-based natural localization is combined with dead reckoning, the particle filter algorithm is commonly applied. Sensor fusion methods commonly involve integrating LiDAR or camera-based techniques with dead reckoning, or combining data obtained from multiple dead reckoning sources, such as rotary encoders and IMU.

There are few publications that are focused on the comparison of localization systems in terms of their accuracy and performance [[46], [47], [48], [49], [50]]. Those studies include research on various implementations of Kalman filter, examination of open-source algorithms running in Robot Operating System, and study on the performance of probabilistic methods. The most commonly used metrics for comparison are mean absolute error or root-mean-square error.

The purpose of this paper is to introduce and evaluate the performance of the dead reckoning based on encoders mounted on the additional passive wheels. This new system is compared with of other AGV localization systems: dead reckoning based on rotary encoders mounted on motors of the drive wheels, dead reckoning based on fused encoders and AHRS data, and approach based on LiDAR natural localization fused with all kinds of dead reckoning techniques mentioned above. In addition, for LiDAR localization two different algorithms were used. In case of LiDAR-based techniques, there is also a possibility of using the LiDAR odometry methods, such as scan matching. However, in this paper only fused LiDAR-based methods are considered, as the relevant research shows, that those methods result in better outcomes [51]. The test scenarios include five different paths: straight line with turning, straight line without turning, spinning in place, square-shaped and eight-shaped track.

## 2 Methods

**Fig. 1:**
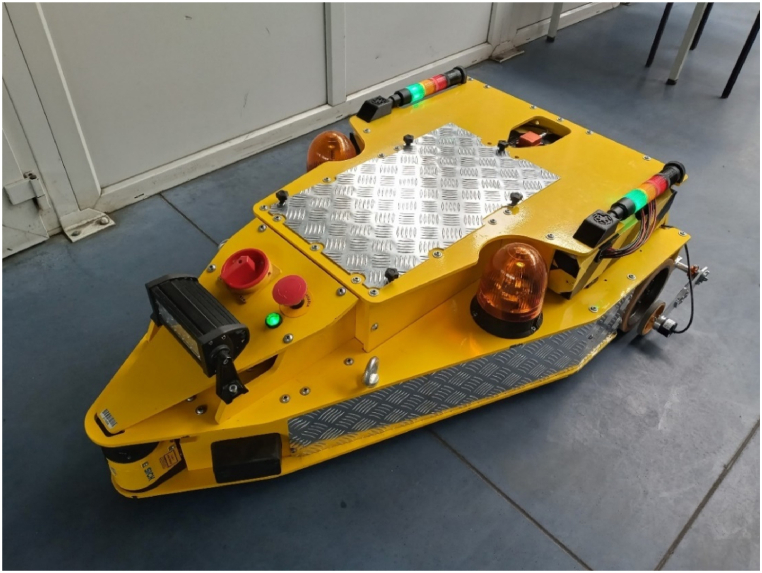
Custom-made automated guided vehicle from the Institute of Mechanical Technology, Poznan University of Technology.

All mentioned localization systems were implemented in autonomous vehicle (Fig. 1) that was designed and manufactured in the Institute of Mechanical Technology (Poznan University of Technology). The AGV is a differential kinematics vehicle with two independent 440 W brushless DC motors and a single passive wheel fixed at its front end. Torque from the engine is transmitted to the wheel through a planetary gearbox and a belt transmission. The construction was made of 8 mm and 12 mm thick steel plates. The weight of the vehicle is about 300 kg. It is equipped with 48 V lithium-ion battery.

**Fig. 2:**
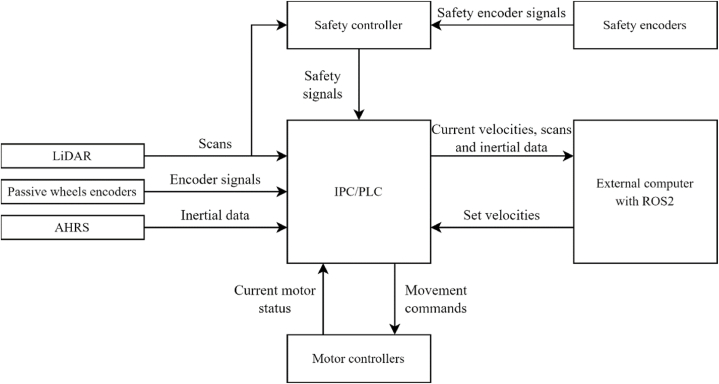
The control system of the automated guided vehicle.

The control system (Fig. 2) consists of two mcDSA-E45 DC motor controllers (miControl, Germany), CX2030 IPC with PLC capabilities (Beckhoff, Germany) and safety controller (SICK, Germany) with S300 Pro LiDAR scanner and DFS60S safety encoders. The main purpose of IPC is the low level control of lamps and sound indicators, communication with motor controllers using CAN and reading data from LiDAR and IMU using RS-422 and USB. The navigation system was implemented using ROS2 navigation stack [52]. The system is running on external computer with an Ubuntu 22.04 OS.

The AGV is equipped with sensors that allow implementation of various localization methods. Those sensors are: incremental encoders mounted on shafts of drive motors, incremental encoders mounted on additional passive wheels, Mti-300 AHRS (Xsens, Netherlands) and LiDAR scanner. Localization systems were implemented mostly using an open-source packages, available in ROS. Although for collecting data from LiDAR and IMU, custom programs were developed using Python and C++.

The use of incremental encoders allows implementing a dead reckoning localization, based on a kinematic model of vehicle. Both linear and angular velocities are calculated using the following formulas:

(2.1) $$\begin{array}{c} v=R\frac{n_{r}+n_{l}\;}{2}∙\frac{2\pi }{60}∙\frac{1}{i}\text{,} \end{array}$$

(2.2) $$\begin{array}{c} \omega =R\frac{n_{r}-n_{l}\;}{L}∙\frac{2\pi }{60}∙\frac{1}{i}\text{,} \end{array}$$

where:

$n_{l}$, $n_{r}$ - wheels rotation speed [RPM],

v - linear velocity [m/s],

ω - angular velocity [rad/s],

L - distance between centers of wheels [m],

R - wheel radius [m],

i - transmission ratio between wheel and motor.

Based on calculated velocities, the global position and orientation of vehicle can be obtained using the formulas:

(2.3) $$\begin{array}{c} \theta =\sum \left(\omega ∙\Delta t\right)\text{,} \end{array}$$

(2.4) $$\begin{array}{c} x=\sum \left(v∙\cos\left(\theta \right)∙\Delta t\right)\text{,} \end{array}$$

(2.5) $$\begin{array}{c} y=\sum \left(v∙\sin\left(\theta \right)∙\Delta t\right)\text{,} \end{array}$$

where:

x – global position in X axis [m],

y – global position in Y axis [m],

θ – global orientation on XY plane [rad],

Δt – time step [s].

**Fig. 3:**
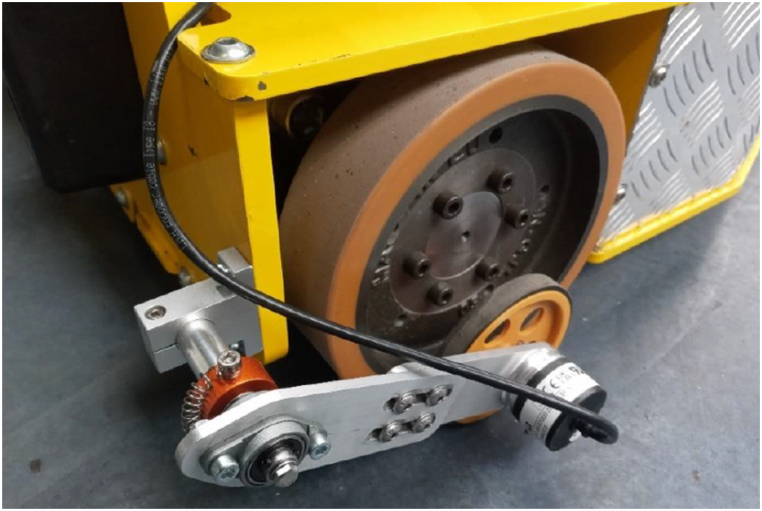
Custom-made passive wheel module as mounted on the vehicle.

Above mentioned dead reckoning localization system was implemented using encoders mounted on two types of wheels: driven and passive. The purpose of using additional passive wheels is to reduce effects of lateral and longitudinal slippage, associated to drive wheels during accelerating, decelerating, and turning. The passive wheel module is presented in Fig. 3. Beside wheel, it includes an encoder for position reading and spring, which function is to ensure constant pressure of the wheel to the ground. There were two passive wheels, one per each side.

**Fig. 4:**
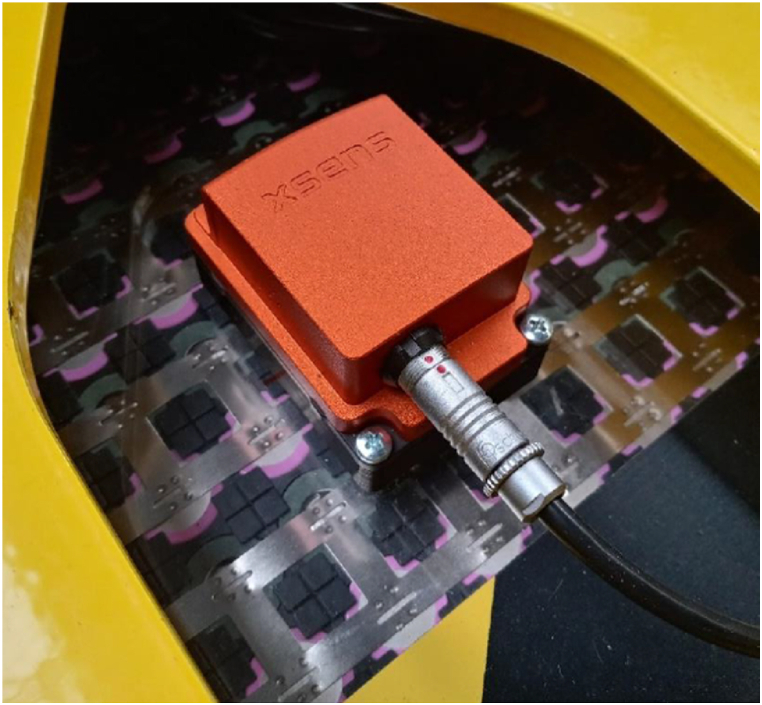
The attitude and heading reference system mounted on vehicle.

The AHRS (Fig. 4) used in vehicle feeds the localization system with data on linear acceleration, angular velocity and global orientation of vehicle. It is mounted in the AGV's rotation axis, so an angular velocity and orientation measured by AHRS matches current angular velocity and orientation of the AGV. Unlike wheels dead reckoning technique, data collected with AHRS cannot be used for global position calculation due to relatively high errors and drift of measurements. In that case, AHRS data was used as one of the inputs of Kalman filter, which purpose is to fuse data obtained from AHRS and wheels dead reckoning. This approach leads to reduced errors related to slippage of vehicle's wheels. The filter was implemented using *„robot_localization”* ROS2 package [53]. The Kalman filter configuration file is available in the attached supplementary materials.

The LiDAR scanner is mounted on the front of the vehicle, so it can correctly perform a safety functions. It also acts as a source of data for LiDAR-based algorithms. The scanner's field of view is 270°. The data obtained by LiDAR was used to prepare a map of vehicle's working area. The map was generated using SLAM algorithm implemented in *„SLAM Toolbox”* ROS2 package [54]. It is used as an absolute reference for LiDAR-based localization system, which was implemented using two different algorithms: AMCL and graph-based. Both algorithms are available in ROS2 navigation stack and *„SLAM Toolbox”* package respectively. Configuration files of both algorithms are available in the attached supplementary materials.

The studied localization systems are combinations of the abovementioned methods and algorithms. Each system includes one source of dead reckoning data (drive or passive wheels), uses or not fused AHRS data, and uses one of two types of LiDAR-based algorithms or neither of them. This results in a total of 12 tested configurations.

**Fig. 5:**
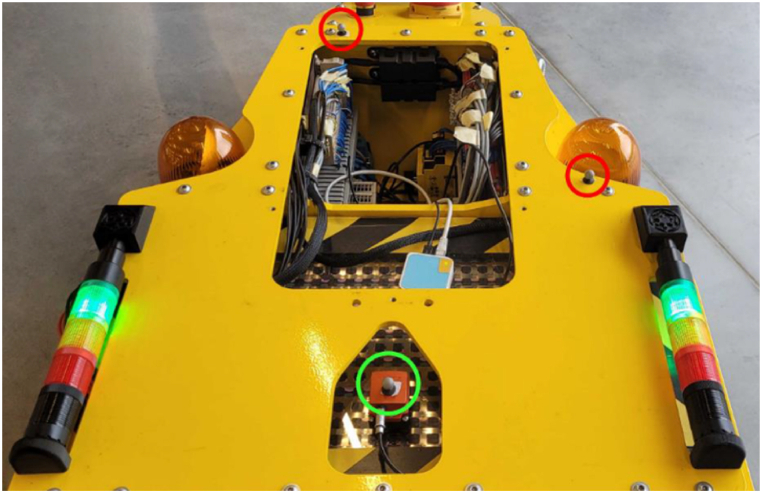
Markers mounted on the automated guided vehicle.

The reference localization of the AGV during experimental research is obtained by OptiTrack motion capture system. The OptiTrack system configuration used in the study consists of twelve „Prime^X^ 13” cameras and the main unit. The operation of these cameras is based on detecting infrared light reflected or generated by the markers, from which the image is generated. These images are then processed within each camera. The processing involves determining the position of each detected marker on the screen matrix and sending this information to a central unit with installed software, where all the data is fused. The result is the absolute position and orientation of the detected markers. The markers mounted on the AGV are presented in Fig. 5. The one marked with green circle represent the reference point for determining the AGV orientation.

## 3 Scenarios

**Fig. 6:**
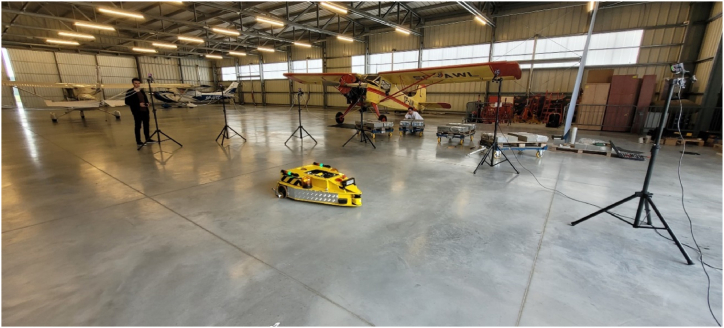
Experimental setup.

The tests were conducted at the University airport hangar located in Kąkolewo, Grodzisk Wielkopolski, Poland. The experimental setup is presented in Fig. 6. The workspace of the OptiTrack system is around 8 m per 6 m. The ground surface was prepared in such a way as to minimize the occurrence of slippages. The environment lacks of unique geometric features, which are important for LiDAR-based algorithms.

There were five scenarios tested, which were designed to measure accuracy of localization systems i.e. to determine either its position or orientation error, or both of them. Each of presented scenarios was performed twice to prevent errors related to unexpected system failure and errors in recorded data.

Scenario #1 includes driving on a straight path with half-turn and return to the start point. The goal is to verify how the capability of each system to determine the global localization of the AGV is affected by making a single turn. Scenario #2 is similar, but the AGV moves in reciprocating motion, without making any turns. That task was performed to verify how accurate the localization systems are in case of determining AGV's position.

In scenario #3, the AGV perform ten full in place rotations. The goal is to investigate how accurate the localization systems are in case of determining an orientation of the AGV. The last two scenarios assume paths similar to the ones, that can be encountered in real industrial environments. The first of them (scenario #4) is square-shaped, and the second is eight-shaped path (scenario #5). The goal of the former is to examine how well the localization systems perform on path assembled from multiple turns and straight lines. The goal of the latter is to verify the accuracy of localization systems during dynamic ride, where linear and angular motions of the AGV are changing at the same time.

The accuracy of presented localization systems was calculated as the arithmetic mean of absolute errors for all given time steps and for each axis along which the AGV was moving. In addition, beside arithmetic mean, a standard deviations for each axis were calculated, to verify the consistency of data generated by implemented localization systems.

**Fig. 7:**
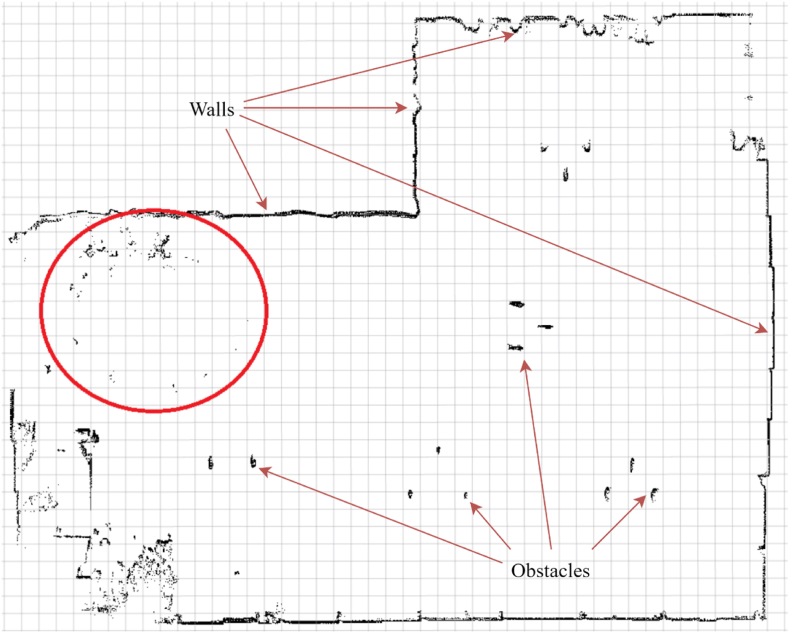
The map of a hangar with the workspace marked with red ellipse. (For interpretation of the references to colour in this figure legend, the reader is referred to the Web version of this article.)

To implement LiDAR-based methods, a map of workspace is required. To prepare the one, which was used during experiment, SLAM algorithm available in *„SLAM Toolbox”* ROS2 package was used. The map (Fig. 7) represents the entire hangar, however the workspace in which the experiment took place is marked with red ellipse. The grid size of the map is 1 m.

## 4 Results

**Fig. 8:**
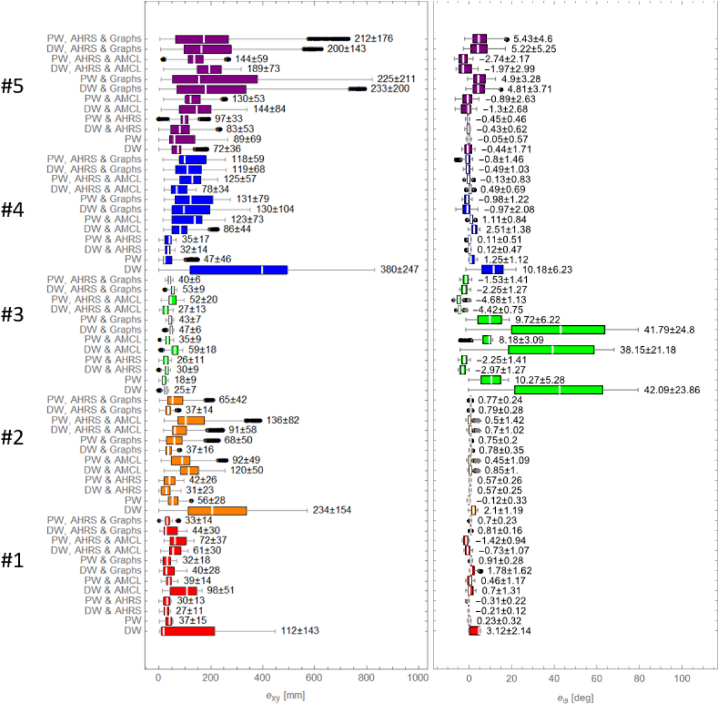
Box and whisker plot presenting the $e_{xy}$ and $e_{\vartheta }$ error distribution. Please note that white line depicts median and numbers indicate mean ± standard deviation values.

The results of experiments are presented in form of box and whisker plots (Fig. 8), which visualize distribution of errors on XY plane and orientations errors, for all considerated cases. The white line on the plots is median. The values indicate mean ± standard deviation. The acronyms DW and PW stand for: DW – drive wheels dead reckoning, PW – passive wheels dead reckoning.

The results of scenario #1, which was a straight path with one half-turn, show that the highest values of error were obtained with standalone drive wheels dead reckoning. Experiment shows, that change of dead reckoning source from drive wheels to passive wheels, or fusing an inertial data from AHRS significantly reduces both position and orientation errors. The results obtained with LiDAR-based methods indicate, that in short term they perform better than standalone drive wheels dead reckoning, but worse that passive wheels dead reckoning. Fusing AHRS with dead reckoning and graph-based technique seemed to slightly reduce the orientation errors. There is no visible improvement in case of AMCL. Generally, graph-based algorithm seems to perform better in that scenario than AMCL.

The results of scenario #2, which was reciprocating motion on straight path, presents similar outcomes, as the ones obtained in first scenario. The highest values of errors correspond to standalone drive wheels dead reckoning. Appliance of passive wheels dead reckoning contribute to remarkable reduction of both types of measured errors. The fused LiDAR-based methods show mediocre results. Again, graph-based algorithm generally feature with lower position errors values than AMCL. Integration of AHRS seemed to have no effect on reducing the values of errors, due to lack of angular motion in that scenario.

In scenario #3, which was spinning in place, there will be considered only orientation errors, as the AGV was not commanded to perform any linear motion. The worst results were obtained with methods, where drive wheels dead reckoning was used, excluding cases, where AHRS was adapted. Change of dead reckoning source from drive wheels to passive wheels results in significant decrease of orientation error. However, the best results are obtained with methods, where AHRS is used. The use of LiDAR-based methods fused with dead reckoning only seemed to have barely any effect on lowering the measured errors.

In scenario #4, which was driving on square-shaped path, the results seem to be very consistent. Again, the worst performance corresponds to standalone drive wheels dead reckoning. The best short-term outcomes result from adapting a standalone passive wheels dead reckoning and fusing dead reckoning with inertial data. The mediocre results were obtained with all methods, where LiDAR-based algorithms were involved. Although, fusing AHRS data alongside LiDAR data results in lower the orientation errors.

The last scenario, where dynamic drive on eight-shaped path was performed, presents outlier results, in compare to all earlier scenarios. The lowest values of errors are obtained by standalone dead reckoning and dead reckoning fused with inertial data, while the worst are obtained with all LiDAR-based methods. Fusion of AHRS with dead reckoning and LiDAR-based techniques also did not bring significant improvement on localization accuracy. In previous scenarios the graph-based algorithm performed better than AMCL, however in the last scenario, AMCL achieved lower values of errors, than graph-based algorithm.

**Fig. 9:**
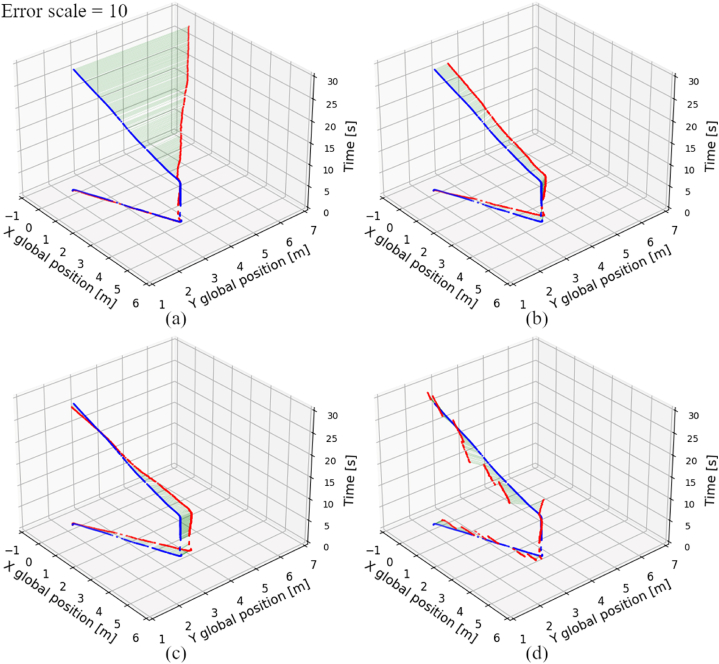
Global position obtained by analyzed localization systems (red) with reference to OptiTrack (blue) in scenario #1, a) drive wheels dead reckoning, b) passive wheels dead reckoning, c) fused drive wheels dead reckoning and AHRS, d) fused drive wheels dead reckoning and graph-based algorithm. (For interpretation of the references to colour in this figure legend, the reader is referred to the Web version of this article.)

In Fig. 9, the global position appointed by certain localization methods in scenario #1 is presented. It is clearly visible, that in case of standalone dead reckoning techniques, the position errors accumulate in time. The value of error rises rapidly after making a turn. The reason of that outcome is that the global position calculation depends on an orientation angle. By making a turn, the orientation error rises, and so does the position error. The results of tested scenario show, that the standalone passive wheels dead reckoning performs similar to the drive wheels dead reckoning fused with AHRS. Both of those techniques perform much better, than standalone drive wheels dead reckoning, due to reduced influence of slippages. In case of fused LiDAR-based techniques, the position error does not rise steady in time, but rather varies around a certain value. That behavior is caused by referencing to a fixed map. The errors related to a mapping process result in an unintended position offset in both X and Y axes.

**Fig. 10:**
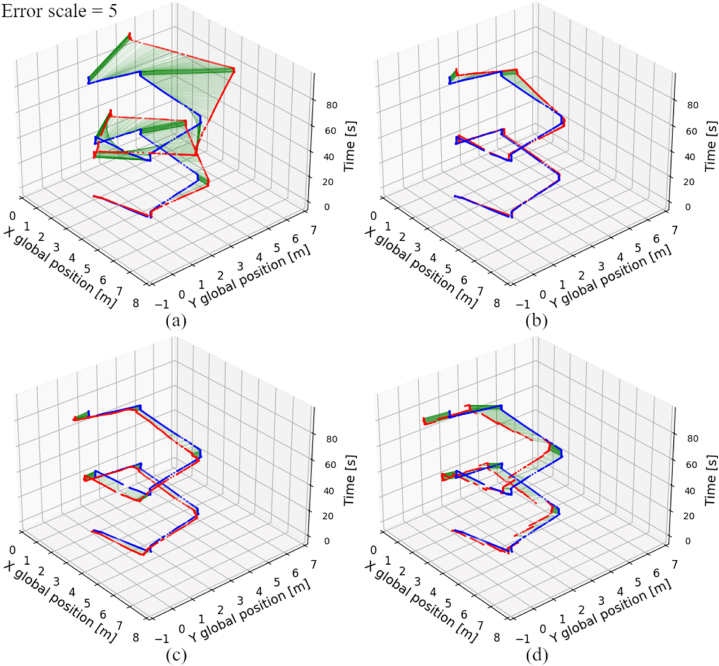
Global position obtained by analyzed localization systems (red) with reference to OptiTrack (blue) in scenario #4, a) drive wheels dead reckoning, b) passive wheels dead reckoning, c) fused drive wheels dead reckoning, AHRS and AMCL, d) fused drive wheels dead reckoning, AHRS and graph-based algorithm. (For interpretation of the references to colour in this figure legend, the reader is referred to the Web version of this article.)

Fig. 10 presents the global position obtained by certain localization techniques in scenario #4. Similarly to results of scenario #1, the position errors appointed by both standalone dead reckoning techniques also seem to steadily rise in time. Again, usage of passive wheels for dead reckoning has a significant influence on reducing localization errors, compared to the same technique, but with usage of the drive wheels. The outcomes of fused LiDAR-based methods in scenario #4 show, that those techniques result in a position offset, which value varies depending on the direction of the AGV movement. The position offset varies, because when the AGV changes its orientation angle, the localization system reference also changes. The different parts of the map are fraught with different values of error, thus the position error of LiDAR-based localization systems depend on current location and orientation of the AGV.

**Fig. 11:**
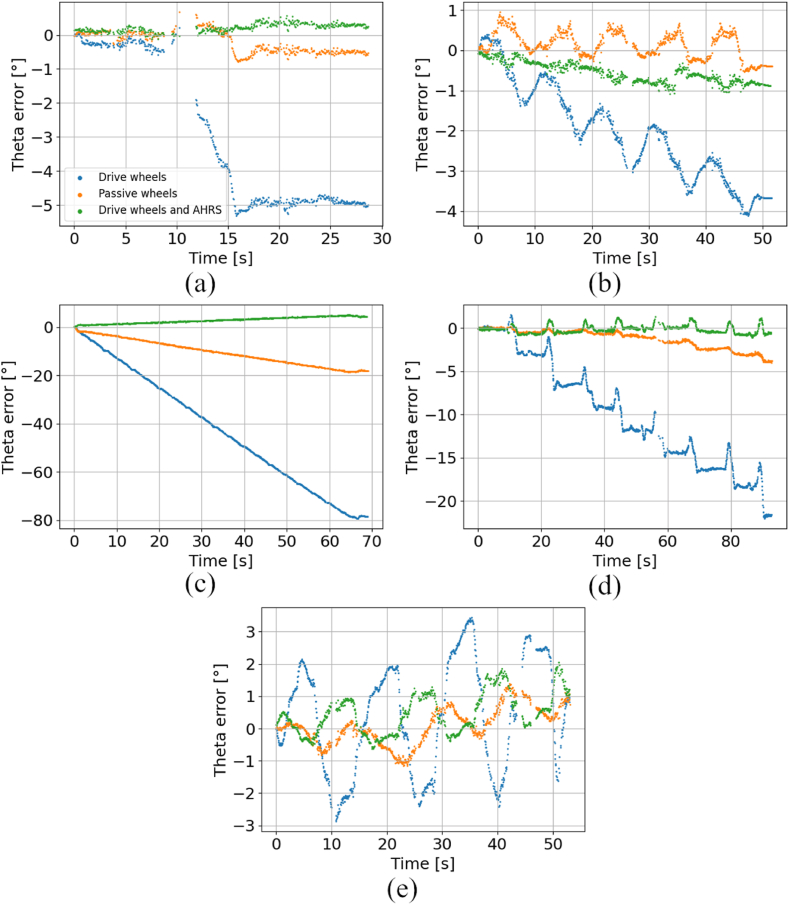
Comparison of orientation errors for dead reckoning techniques and fused AHRS method, a) scenario #1, b) scenario #2, c) scenario #3, d) scenario #4, e) scenario #5.

In Fig. 11, the courses of orientation errors obtained by certain localization methods in all five scenarios are presented. Similarly to position errors, the orientation errors of dead reckoning techniques constantly rise in time. The orientation error rises faster in scenarios, where more angular movement is performed. In case of the orientation errors, the performance of passive wheels dead reckoning is similar to drive wheels dead reckoning fused with AHRS. Identical outcome was obtained, when position errors were compared. The discontinuity in scenario #1 is caused by lack of reference data in a certain timestamps.

**Fig. 12:**
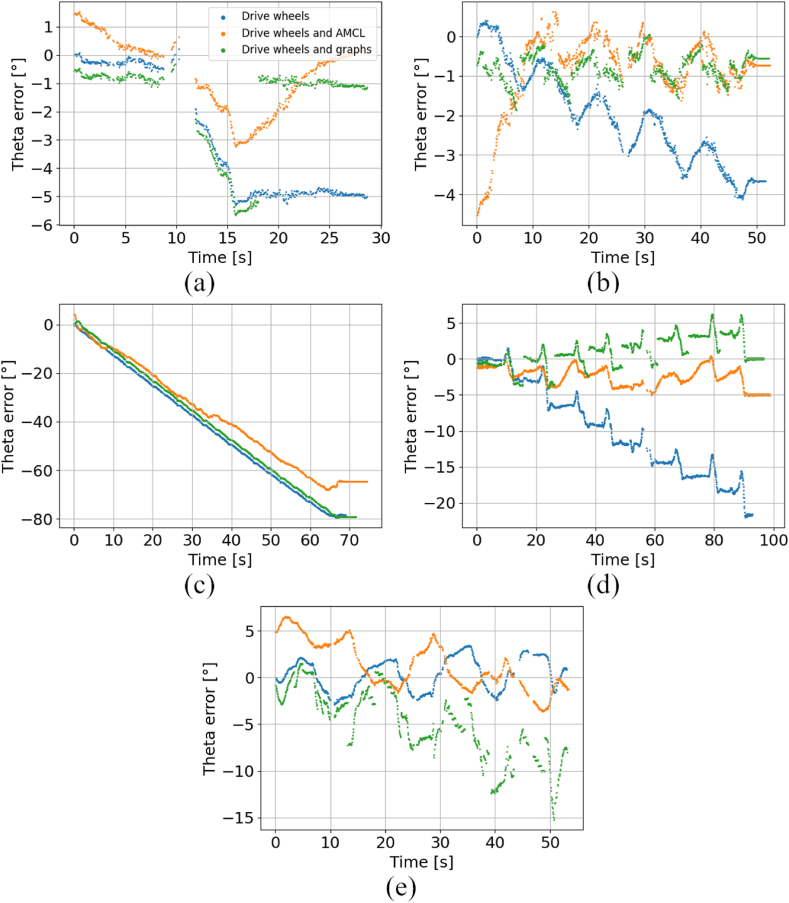
Comparison of orientation errors for fused LiDAR-based techniques and drive wheels dead reckoning, a) scenario #1, b) scenario #2, c) scenario #3, d) scenario #4, e) scenario #5.

Fig. 12 presents the comparison of orientation error between standalone drive wheels dead reckoning and LiDAR-based techniques in all five scenarios. The results indicate, that in most cases, the orientation error of LiDAR-based techniques is reduced. Instead of a steady rise of error, these techniques characterize with error varying around a certain value. However, there were scenarios #3 and #5, where due to dynamic motion of the AGV, the LiDAR-based localization algorithms were not able to properly operate. More results can be found in the supplementary materials to this article.

## 5 Discussion

The result shows, that in every scenario, excluding scenario #5, passive wheels dead reckoning performed much better than drive wheels dead reckoning. The reason of that outcome are reduced lateral and longitudal slippages. In most cases, the integration of AHRS with drive wheels dead reckoning makes the errors values reduced to the ones obtained with standalone passive wheels dead reckoning. However, combining passive wheels dead reckoning with AHRS does not result in significant error reduction. The impact of AHRS integration is particularly evident, when besides of linear motion, a lot of angular motion is performed. In scenario #5, the results obtained with both standalone dead reckoning methods seem to be alike. The cause of an outlier results in that scenario is the symmetrical shape of a path, which makes the cumulative positioning error compensating each time the AGV is changing the direction of movement. Even the integration of AHRS in that particular scenario did not bring much reduction in case of both position and orientation errors.

However, in other scenarios, the fused LiDAR-based methods seem to bring mediocre results. Combining LiDAR data with drive wheels dead reckoning results in reduction of position and orientation errors, which impact is varying of particular scenario. On the other hand, fusion of LiDAR-based methods with passive wheels dead reckoning worsens the accuracy of localization algorithms. Angular motion of the AGV seem to have bad influence on the performance of fused LiDAR-based methods. In most cases, the additional integration of AHRS slightly improves the performance of those techniques. The relevant studies show, that the use of LiDAR data in fused methods gives better results, than using it for the standalone LiDAR odometry [51,55].

Conner et al. showed that adding additional sensing on the castor can improve the localization performance of the dead reckoning [45]. Same observation can be made in our work. That previous study involve mounting the sensor on rear castor wheel which behavior is hardly predictable especially when changing the motion direction in place as in scenario #2 and #4. In those scenarios passive wheel exhibit superior performance in comparison to dead reckoning based on drive wheels only. It also show similar results when compared to other more expensive localization systems.

Despite of greater mean errors values of LiDAR-based methods, for most scenarios the errors have a constant trend, while for other methods, they seem to constantly rise in time. The only scenarios, where errors corresponding to LiDAR-based techniques are frequently rising are third – in place rotations, and fifth – eight-shaped path. It is caused by lack of possibility for localization algorithm to match current LiDAR data with the map, due to dynamic movement of the AGV. The constant trend of errors related to LiDAR-based methods implies an offset, which was added to position and orientation by localization algorithms. Poor accuracy of those result from lack of valid reference data on a map – almost empty hangar on the right side and open gate on the left side of the map, presence of trailers around the workspace area during tests, undefined shapes (noise) in the workstation area and changed arrangement of the curtain next to the workspace.

There are quite few publications relating to the comparison of this many localization systems and its combinations. A well-studied technique is UWB (Ultra Wide Band). There were approaches based on both standalone UWB and its fusion with inertial navigation system [[25], [56], [57]]. The results showed that integration of inertial data into localization system could lead to lower localization errors, especially orientation errors. The similar outcomes were obtained in presented study. Another well studied approaches are various Kalman filter implementations, where a variety of incremental (dead reckoning, inertial data) and absolute (LiDAR-based techniques, UWB) localization techniques are fused [54,[55], [58], [59], [60]]. The obtained results also indicate a positive influence of AHRS on AGV localization system accuracy. A further studies show, that combining incremental and absolute localization techniques leads to minimalization of cumulative errors [61,62]. The similar conclusions stem from the presented study.

In summary, in short-term the best performing techniques are passive wheels dead reckoning and dead reckoning fused with AHRS. As expected, standalone drive wheels dead reckoning is not a reasonable application due to large values of both position and orientation errors, practically in every scenario. The best long-term methods are fused LiDAR-based techniques, especially graph-based method, due to constant value of errors. The accuracy of those is highly correlated with the quality of prepared map – the more unique shapes presence on the map, the better the quality is. If the AGV is performing a lot of angular motion, the AHRS integration is recommended. In case of path's shape, it is better, when it consists of straight lines and in place rotations, like in scenario #4, rather than combination of those movements, like in scenario #5.

## 6 Conclusion

This paper presents the research results on localization accuracy of various AGV localization techniques, including dead reckoning with two types of wheels, fused AHRS and fused LiDAR-based algorithms and its combinations. Presented localization methods were implemented in custom differential drive AGV, manufactured by the authors of this paper. The experiment concludes an outcomes obtained in five scenarios, where various paths and combinations of movements were considered. The research shows the major differences between drive wheels dead reckoning and the newly proposed passive wheels dead reckoning, the influence of fusing inertial data obtained by AHRS and the impact of fusing one of two types of LiDAR-based algorithms with standalone dead reckoning or fused dead reckoning and inertial data. The performance of the localization was tested in five different scenarios. It was shown that dead reckoning using passive wheels is a valuable alternative to other localization system.

The main findings of this research can be summarized in the following bullet points:

• for scenario #1, which was a straight path with one half-turn, the best methods were dead reckoning techniques fused with AHRS data;
• for scenario #2, which was reciprocating motion on straight path, the lowest errors were obtained also by dead reckoning techniques fused with AHRS data;
• for scenario #3, which was spinning in place, the best results were acquired by all techniques, where AHRS was involved;
• for scenario #4, which was driving on square-shaped path, the lowest errors were achieved by dead reckoning techniques fused with AHRS data;
• for scenario #5, which was driving on eight-shaped path, the best methods were standalone dead reckoning techniques and its fusions with AHRS data. Note, that the results for this scenario are unreliable due to special conditions.

Although economic aspect of implementing certain localization method can always be debatable, authors believe that passive wheels can be considered a cost-effective solution and a valuable alternative when compared to LiDAR- or AHRS-based systems.

## CRediT authorship contribution statement

**Kacper Bereszyński:** Writing – review & editing, Writing – original draft, Visualization, Validation, Software, Methodology, Investigation, Formal analysis, Conceptualization. **Marcin Pelic:** Visualization, Validation, Supervision, Methodology, Formal analysis, Data curation, Conceptualization. **Wojciech Paszkowiak:** Investigation, Formal analysis. **Stanisław Pabiszczak:** Methodology, Investigation. **Adam Myszkowski:** Resources, Methodology, Conceptualization. **Krzysztof Walas:** Validation, Software, Resources. **Grzegorz Czechmanowski:** Validation, Investigation. **Jan Węgrzynowski:** Validation, Investigation. **Tomasz Bartkowiak:** Writing – review & editing, Writing – original draft, Supervision, Resources, Project administration, Conceptualization.

## Declaration of competing interest

The authors declare the following financial interests/personal relationships which may be considered as potential competing interests:Tomasz Bartkowiak reports financial support was provided by Ministry of Education and Science of the Republic of Poland. Tomasz Bartkowiak reports a relationship with Ministry of Education and Science of the Republic of Poland that includes: funding grants. If there are other authors, they declare that they have no known competing financial interests or personal relationships that could have appeared to influence the work reported in this paper.

## References

[1] De Ryck M., Versteyhe M., Debrouwere F. (2020). Automated guided vehicle systems, state-of-the-art control algorithms and techniques. J. Manuf. Syst..

[2] Campbell S., O'Mahony N., Krpalcova L., Riordan D., Walsh J., Murphy A., Ryan C. (2018).

[3] Borenstein J., Everett H.R., Feng L., Wehe D. (1997). Mobile robot positioning: sensors and techniques. J. Rob. Syst..

[4] Feledy C., Luttenberger S. (2017). A state of the art map of the AGVS technology and a guideline for how and where to use it, M.S. Thesis. Dept.

[5] Malagon-Soldara S.M., Toledano-Ayala M., Soto-Zarazua G., Carrillo-Serrano R.V., Rivas-Araiza E.A. (2015). Mobile robot localization: a review of probabilistic map-based techniques. IAES Int. J. Rob. Autom..

[6] Huang S., Dissanayake G. (2016). Wiley Encyclopedia of Electrical and Electronics Engineering.

[7] Campbell S., O'Mahony N., Carvalho A., Krpalkova L., Riordan D., Walsh J. (2020). 2020 6th International Conference on Mechatronics and Robotics Engineering.

[8] Ronzoni D., Olmi R., Secchi C., Fantuzzi C. (2011). Proc IEEE Int Conf Robot Autom.

[9] Yilmaz A., Temeltas H. (2019). Self-adaptive Monte Carlo method for indoor localization of smart AGVs using LIDAR data. Robot. Autonom. Syst..

[10] Liu Y., Piao Y., Zhang L. (2021). Research on the positioning of AGV based on lidar. J Phys Conf Ser.

[11] Feng L., Bi S., Dong M., Hong F., Liang Y., Lin Q., Liu Y. (2018). 2017 IEEE 7th Annual International Conference on CYBER Technology in Automation, Control, and Intelligent Systems, CYBER 2017.

[12] Belkin I., Abramenko A., Yudin D. (2021). Procedia Comput Sci.

[13] Liu Y., Wang C., Wu H., Wei Y., Ren M., Zhao C. (2022). Improved LiDAR localization method for mobile robots based on multi-sensing. Remote Sens (Basel).

[14] Fox D., Thrun S., Burgard W., Dellaert F. (2001). Particle filters for mobile robot localization. Sequential Monte Carlo Methods in Practice.

[15] Patruno C., Colella R., Nitti M., Renò V., Mosca N., Stella E. (2020). A vision-based odometer for localization of omnidirectional indoor robots. Sensors.

[16] Isozaki N., Chugo D., Yokota S., Takase K. (2011, 2011). 2011 IEEE International Conference on Mechatronics and Automation.

[17] Liu X., Wang G., Chen K. (2022).

[18] Garcia-Rodriguez A., Castillo-Garcia J.G., Gonzalez-Hernandez H.G., Reyes-Avendano J.A., Mora-Salinas R.J. (2021). ACM International Conference Proceeding Series.

[19] Skrzypczyński P. (2017). Advances in Intelligent Systems and Computing.

[20] Biswas J., Veloso M. (2012). Depth camera based indoor mobile robot localization and navigation. Proc IEEE Int Conf Robot Autom.

[21] Cho H., Song H., Park M., Kim J., Woo S., Kim S. (2013). Proceedings - IEEE International Workshop on Robot and Human Interactive Communication.

[22] Cramer M., Cramer J., de Schepper D., Aerts P., Kellens K., Demeester E. (2020). Procedia CIRP.

[23] Grilo A., Costa R., Figueiras P., Goncalves R.J. (2021). 2021 IEEE International Conference on Engineering, Technology and Innovation, ICE/ITMC 2021 - Proceedings.

[24] Barai S., Biswas D., Sau B. (2017, 2018). 2017 IEEE Conference on Antenna Measurements and Applications.

[25] Shi D., Mi H., Collins E.G., Wu J. (2020). An indoor low-cost and high-accuracy localization approach for AGVs. IEEE Access.

[26] Kirsch C., Röhrig C. (2011). IFAC Proceedings Volumes (IFAC-PapersOnline).

[27] Raghavan A.N., Ananthapadmanaban H., Sivamurugan M.S., Ravindran B. (2010). Proc IEEE Int Conf Robot Autom.

[28] Loevsky I., Shimshoni I. (2010). Reliable and efficient landmark-based localization for mobile robots. Robot. Autonom. Syst..

[29] Gao X., Wang J., Chen W. (2015). 2015 IEEE International Conference on Robotics and Biomimetics.

[30] Zheng Z., Lu Y. (2022). Research on AGV trackless guidance technology based on the global vision. Sci. Prog..

[31] Wu P.L., Li J.J., Shaw J.S. (2022). Development of an omnidirectional AGV by applying ORB-SLAM for navigation under ROS framework. Journal of Automation, Mobile Robotics and Intelligent Systems.

[32] Whitaker T.J.L., Cunningham S.J., Bobda C. (2020). Decentralised indoor smart camera mapping and hierarchical navigation for autonomous ground vehicles. IET Comput. Vis..

[33] Farahan S.B., Machado J.J.M., de Almeida F.G., Tavares J.M.R.S. (2022). 9-DOF IMU-based attitude and heading estimation using an extended kalman filter with bias consideration. Sensors.

[34] Stanculeanu I., Borangiu T. (2012). IFAC Proceedings Volumes (IFAC-PapersOnline).

[35] Jeon D., Choi H., Kim J. (2016). 2016 13th International Conference on Ubiquitous Robots and Ambient Intelligence.

[36] Quan S., Chen J. (2019). Proceedings - 2019 2nd World Conference on Mechanical Engineering and Intelligent Manufacturing.

[37] De Cecco M. (2002). Conference Record - IEEE Instrumentation and Measurement Technology Conference.

[38] Hu X., Luo Z., Jiang W. (2020). AGV localization system based on ultra‐wideband and vision guidance. Electronics (Switzerland).

[39] Ding G., Lu H., Bai J., Qin X. (2020). 2020 5th International Conference on Control and Robotics Engineering.

[40] Temeltas Hakan (2018). A real-time localization method for agvs in smart factories. International Scientific Journal “Science. Business. Society.

[41] Filip I., Pyo J., Lee M., Joe H. (2023). LiDAR SLAM with a wheel encoder in a featureless tunnel environment. Electronics (Switzerland).

[42] Xu H., Li Y., Lu Y. (2023). J Phys Conf Ser.

[43] Cai G.S., Lin H.Y., Kao S.F. (2019). 2019 International Automatic Control Conference.

[44] Cong T.H., Kim Y.J., Lim M.T. (2008). 2008 International Conference on Control, Automation and Systems.

[45] Conner D.C., Kedrowski P.R., Reinholtz C.F., Bay J.S. (2001). Improved dead reckoning using caster wheel sensing on a differentially steered three-wheeled autonomous vehicle.

[46] Ndjeng A.N., Lambert A., Gruyer D., Glaser S. (2009). IEEE Intelligent Vehicles Symposium, Proceedings.

[47] Cho H., Kim E.K., Jang E., Kim S. (2016). Lecture Notes in Computer Science (Including Subseries Lecture Notes in Artificial Intelligence and Lecture Notes in Bioinformatics).

[48] Sankalprajan P., Sharma T., Perur H.D., Sekhar Pagala P. (2020). 2020 International Conference for Emerging Technology.

[49] Kirsch C., Kuenemund F., Hess D., Roehrig C. (2012). VDE Conference Publication | IEEE Xplore, ROBOTIK 2012, 7th German Conference on Robotics.

[50] Conner D.C., Kedrowski P.R., Reinholtz C.F., Bay J.S., Choset H.M., Gage D.W., Stein M.R. (2001). Improved Dead Reckoning Using Caster Wheel Sensing on a Differentially Steered Three-Wheeled Autonomous Vehicle.

[51] Wu Q., Meng Q., Tian Y., Zhou Z., Luo C., Mao W., Zeng P., Zhang B., Luo Y. (2022). A method of calibration for the distortion of LiDAR integrating IMU and odometer. Sensors.

[52] Nav2 — Nav2 ROS2 Package documentation, (n.d.). https://navigation.ros.org/(accessed November 21, 2023).

[53] GitHub - Robot Localization ROS2 Package, (n.d.). https://github.com/cra-ros-pkg/robot_localization (accessed November 21, 2023).

[54] S. Macenski, GitHub - Slam Toolbox ROS2 Package, (n.d.). https://github.com/SteveMacenski/slam_toolbox (accessed November 21, 2023).

[55] Xue H., Fu H., Dai B. (2019). IMU-aided high-frequency lidar odometry for autonomous driving. Appl. Sci..

[56] Shi D., Mi H., Collins E.G., Wu J. (2020). An indoor low-cost and high-accuracy localization approach for AGVs. IEEE Access.

[57] Yudanto R.G., Petre F. (2015). 2015 International Conference on Indoor Positioning and Indoor Navigation.

[58] Yuan P., Chen D., Wang T., Ma F., Ren H., Liu Y., Tan H. (2014). Proceedings - 2014 International Symposium on Computer, Consumer and Control.

[59] Shi E., Wang Z., Huang X., Huang Y. (2009). 2009 IEEE International Conference on Robotics and Biomimetics.

[60] Yuan C., Wang Y., Liu J. (2022). Research on multi-sensor fusion-based AGV positioning and navigation technology in storage environment. J Phys Conf Ser.

[61] Yoon S.W., Park S.B., Kim J.S. (2015). Kalman filter sensor fusion for Mecanum wheeled automated guided vehicle localization. J. Sens..

[62] chun Wang T., sheng Tong C., ling Xu B. (2020). AGV navigation analysis based on multi-sensor data fusion. Multimed. Tool. Appl..

