# Supplementary figures and images for: Passive wheels – A new localization system for automated guided vehicles
Source: Heliyon. 2024 Jul 20;10(15):e34967. doi: 10.1016/j.heliyon.2024.e34967 (PMC11829107; doi:10.1016/j.heliyon.2024.e34967)

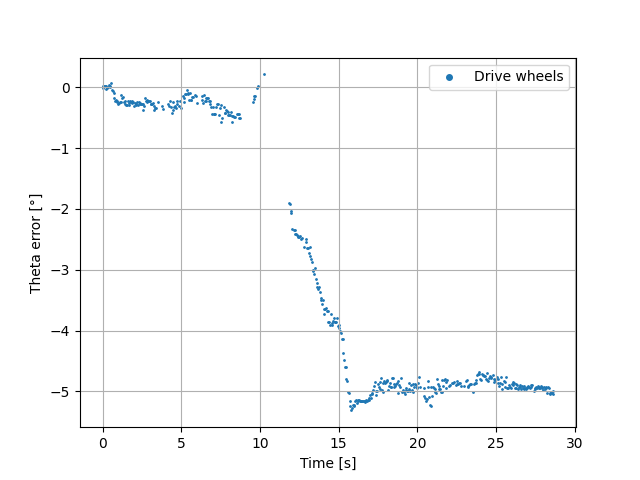

Supplement: Multimedia component 1 [file mmc1.zip › repository/orientation_errors/scenario1_dw.png]

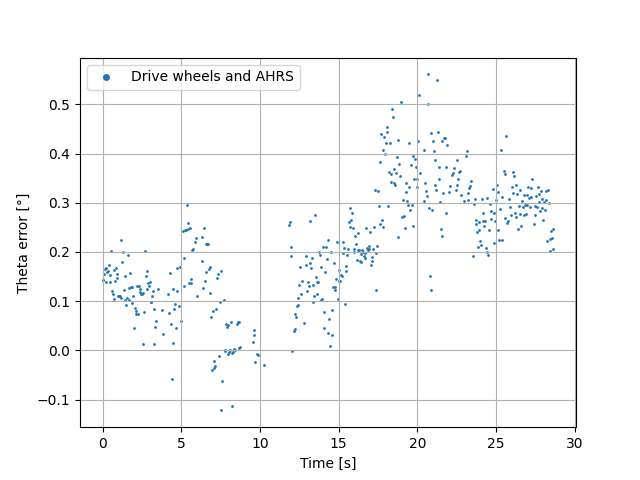

Supplement: Multimedia component 1 [file mmc1.zip › repository/orientation_errors/scenario1_dw_ahrs.png]

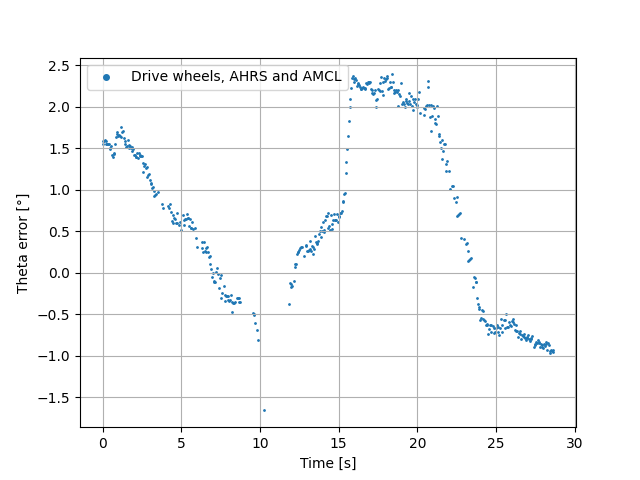

Supplement: Multimedia component 1 [file mmc1.zip › repository/orientation_errors/scenario1_dw_ahrs_amcl.png]

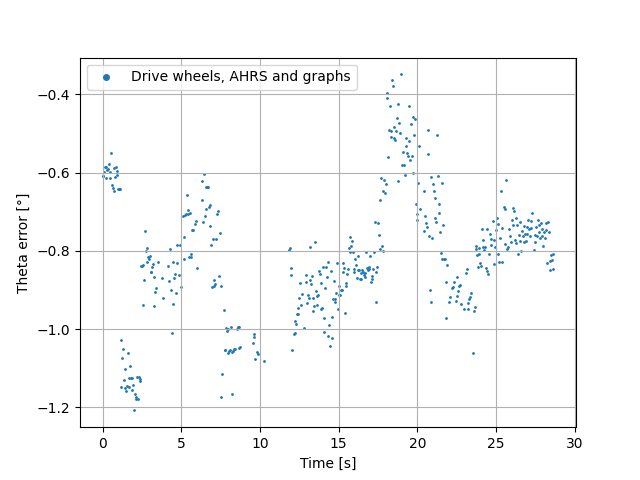

Supplement: Multimedia component 1 [file mmc1.zip › repository/orientation_errors/scenario1_dw_ahrs_graphs.png]

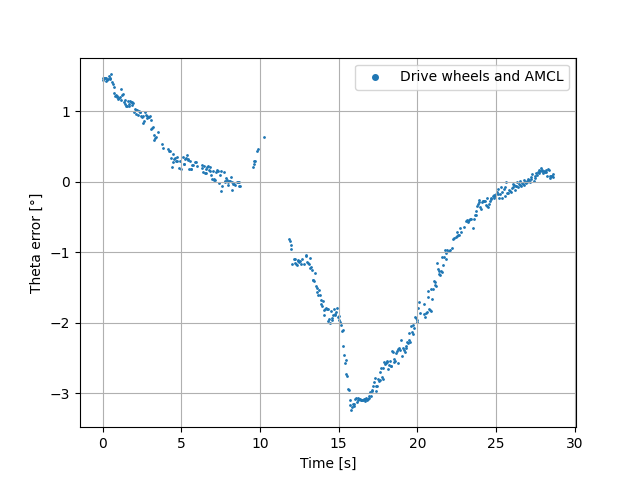

Supplement: Multimedia component 1 [file mmc1.zip › repository/orientation_errors/scenario1_dw_amcl.png]

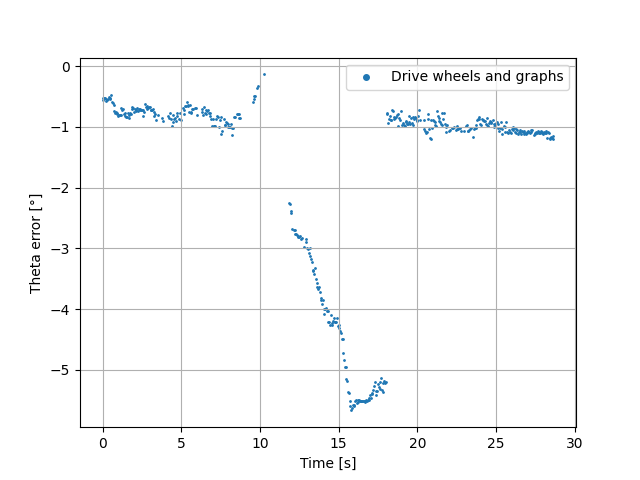

Supplement: Multimedia component 1 [file mmc1.zip › repository/orientation_errors/scenario1_dw_graphs.png]

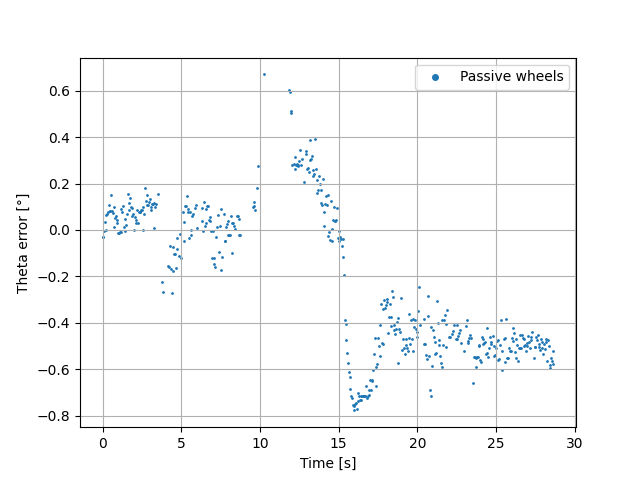

Supplement: Multimedia component 1 [file mmc1.zip › repository/orientation_errors/scenario1_pw.png]

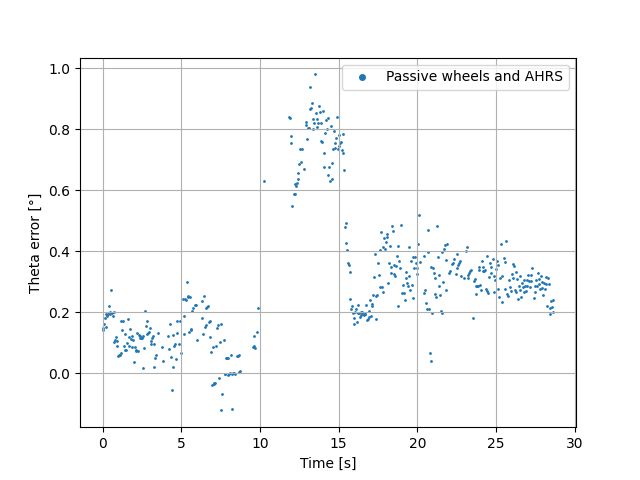

Supplement: Multimedia component 1 [file mmc1.zip › repository/orientation_errors/scenario1_pw_ahrs.png]

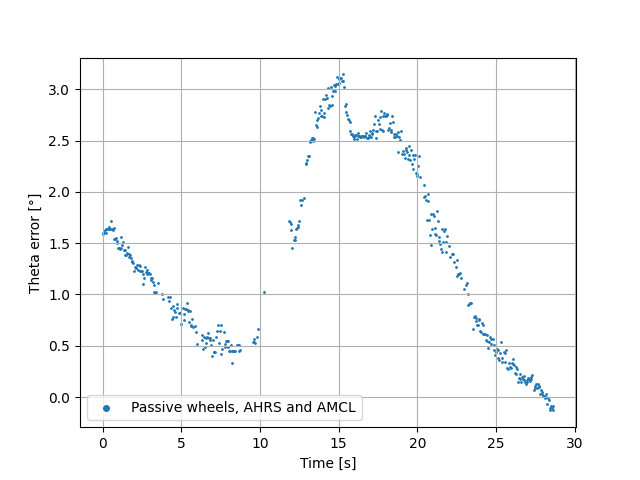

Supplement: Multimedia component 1 [file mmc1.zip › repository/orientation_errors/scenario1_pw_ahrs_amcl.png]

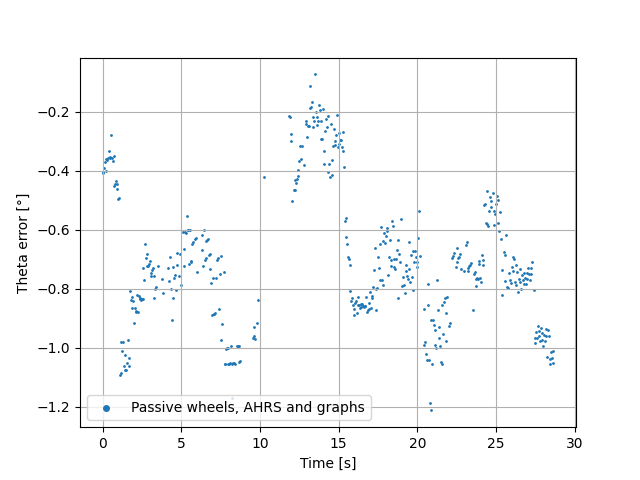

Supplement: Multimedia component 1 [file mmc1.zip › repository/orientation_errors/scenario1_pw_ahrs_graphs.png]

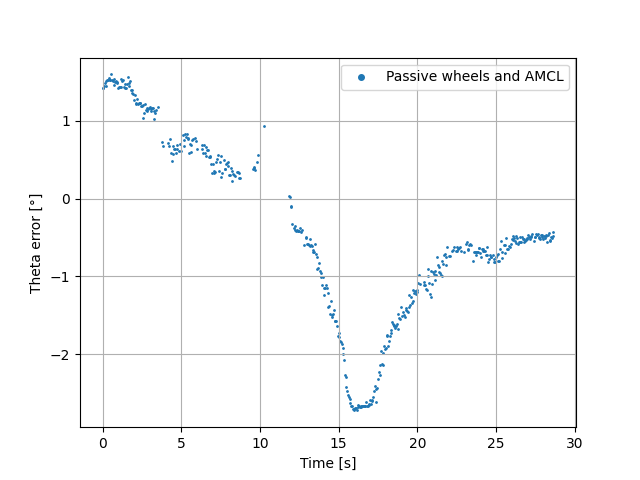

Supplement: Multimedia component 1 [file mmc1.zip › repository/orientation_errors/scenario1_pw_amcl.png]

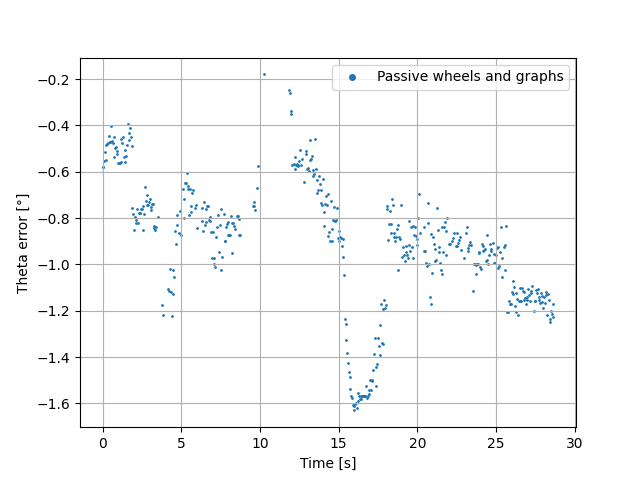

Supplement: Multimedia component 1 [file mmc1.zip › repository/orientation_errors/scenario1_pw_graphs.png]

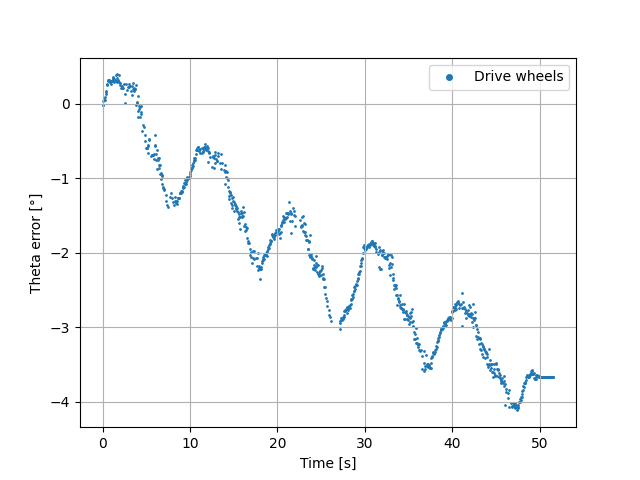

Supplement: Multimedia component 1 [file mmc1.zip › repository/orientation_errors/scenario2_dw.png]

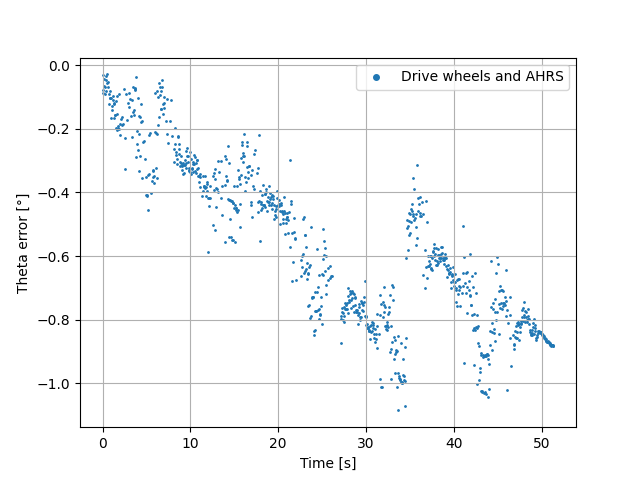

Supplement: Multimedia component 1 [file mmc1.zip › repository/orientation_errors/scenario2_dw_ahrs.png]

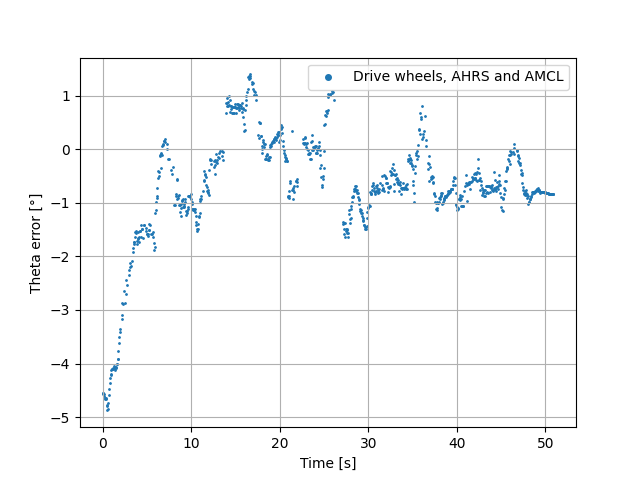

Supplement: Multimedia component 1 [file mmc1.zip › repository/orientation_errors/scenario2_dw_ahrs_amcl.png]

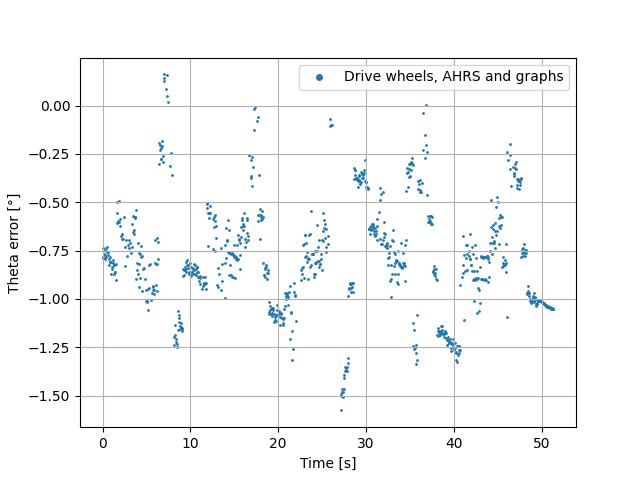

Supplement: Multimedia component 1 [file mmc1.zip › repository/orientation_errors/scenario2_dw_ahrs_graphs.png]

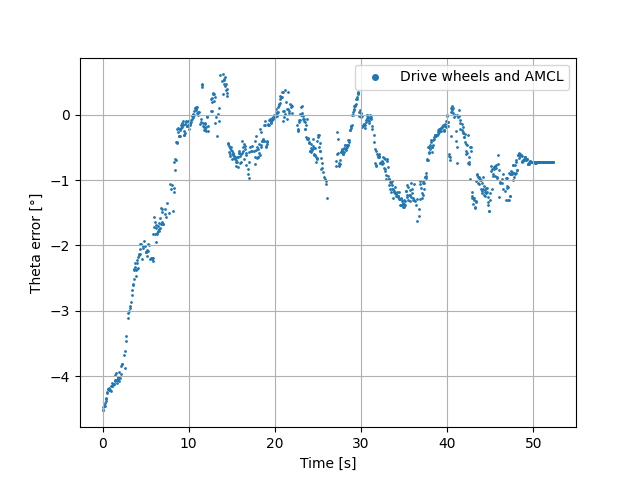

Supplement: Multimedia component 1 [file mmc1.zip › repository/orientation_errors/scenario2_dw_amcl.png]

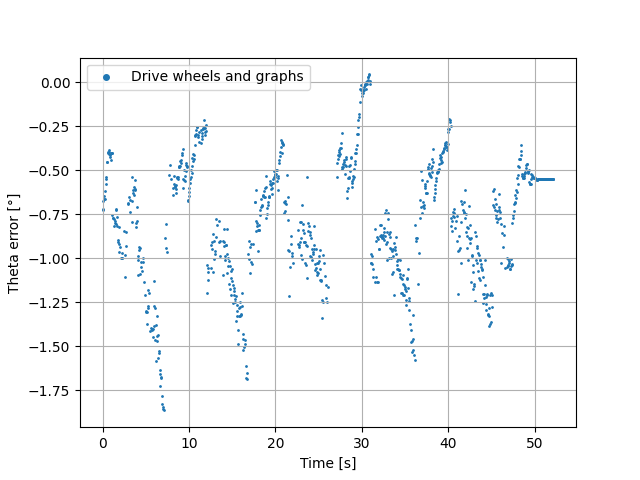

Supplement: Multimedia component 1 [file mmc1.zip › repository/orientation_errors/scenario2_dw_graphs.png]

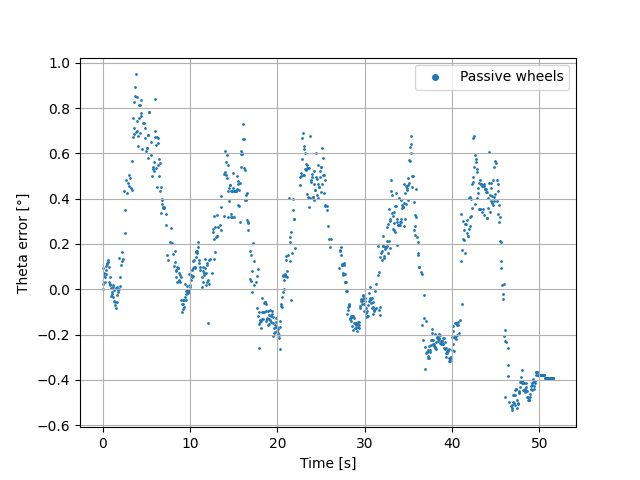

Supplement: Multimedia component 1 [file mmc1.zip › repository/orientation_errors/scenario2_pw.png]

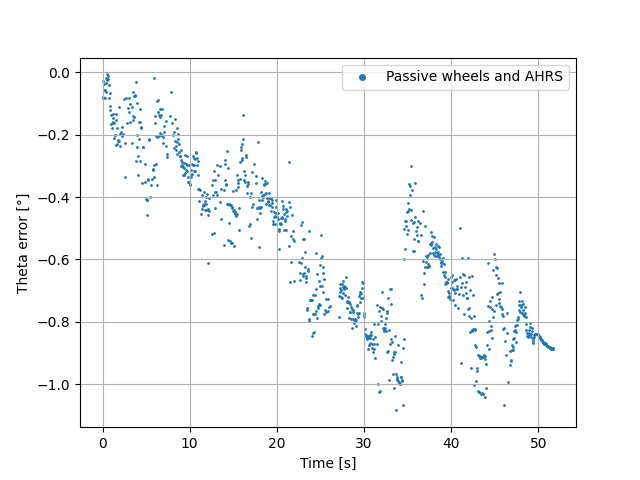

Supplement: Multimedia component 1 [file mmc1.zip › repository/orientation_errors/scenario2_pw_ahrs.png]

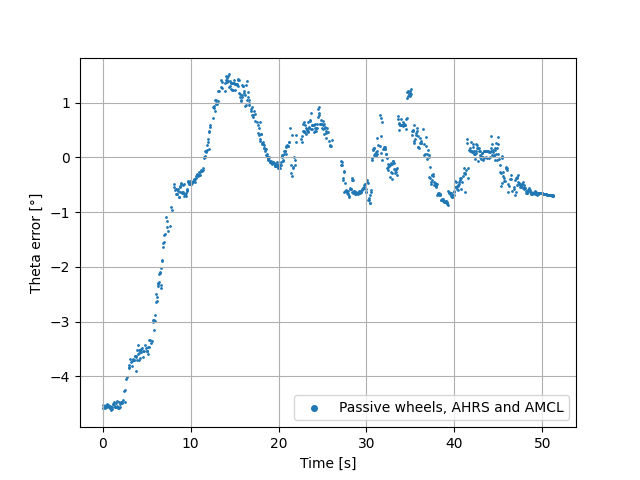

Supplement: Multimedia component 1 [file mmc1.zip › repository/orientation_errors/scenario2_pw_ahrs_amcl.png]

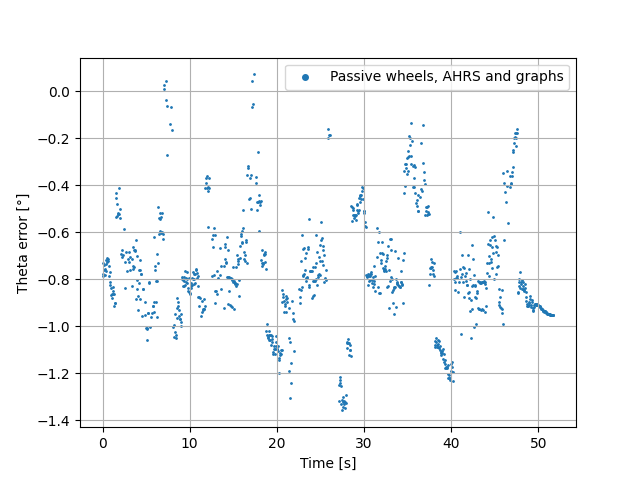

Supplement: Multimedia component 1 [file mmc1.zip › repository/orientation_errors/scenario2_pw_ahrs_graphs.png]

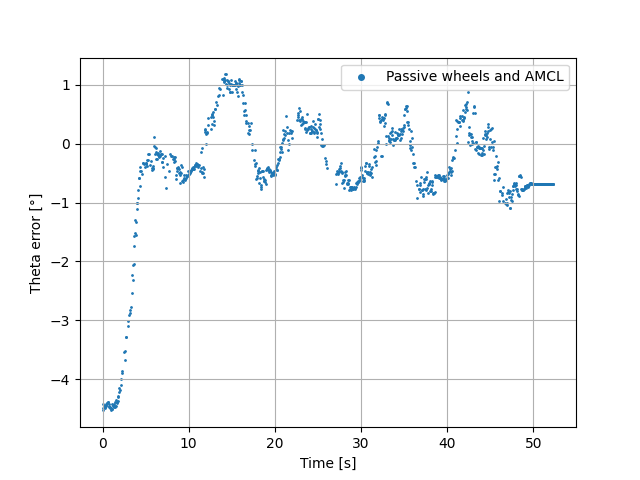

Supplement: Multimedia component 1 [file mmc1.zip › repository/orientation_errors/scenario2_pw_amcl.png]

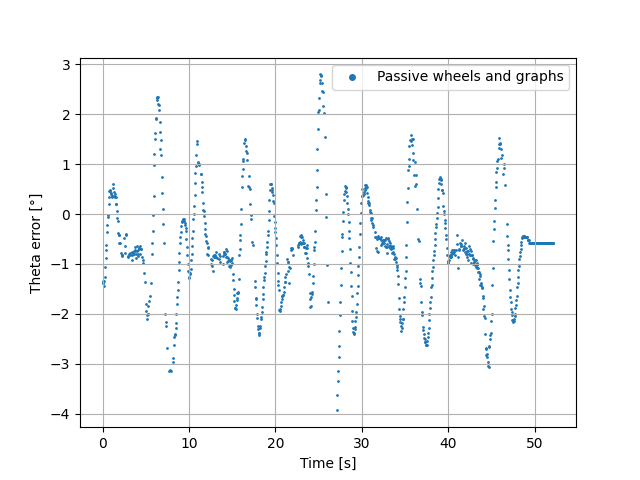

Supplement: Multimedia component 1 [file mmc1.zip › repository/orientation_errors/scenario2_pw_graphs.png]

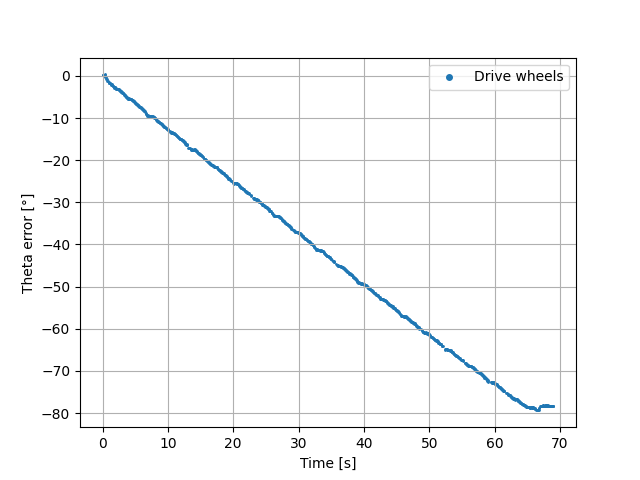

Supplement: Multimedia component 1 [file mmc1.zip › repository/orientation_errors/scenario3_dw.png]

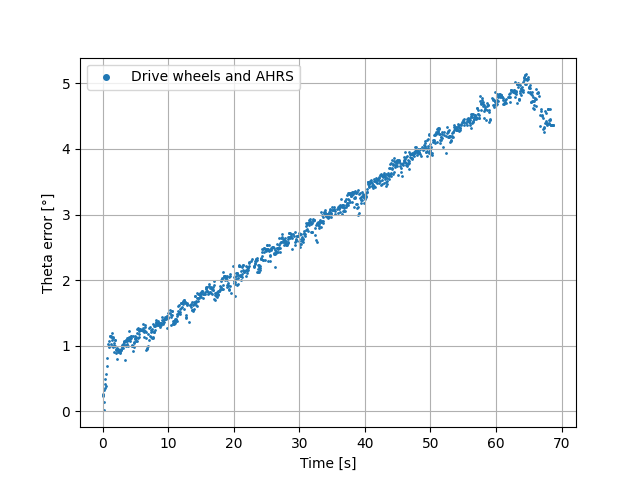

Supplement: Multimedia component 1 [file mmc1.zip › repository/orientation_errors/scenario3_dw_ahrs.png]

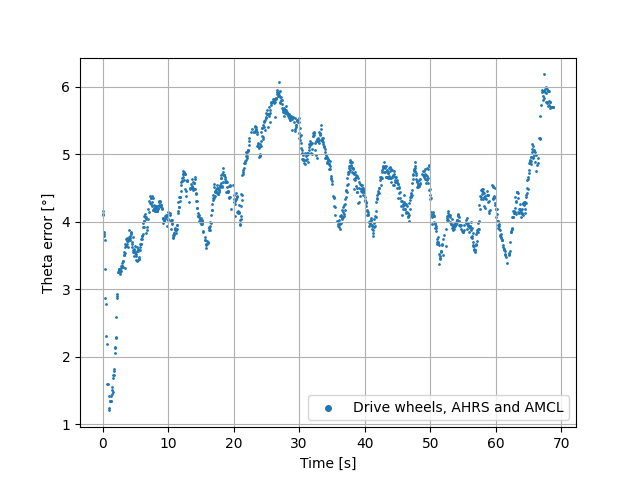

Supplement: Multimedia component 1 [file mmc1.zip › repository/orientation_errors/scenario3_dw_ahrs_amcl.png]

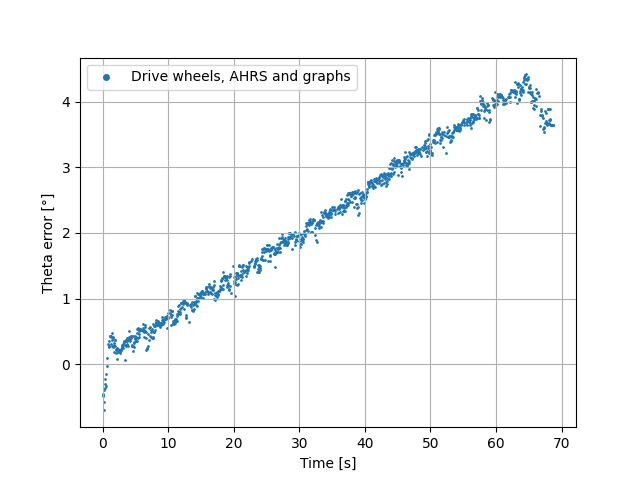

Supplement: Multimedia component 1 [file mmc1.zip › repository/orientation_errors/scenario3_dw_ahrs_graphs.png]

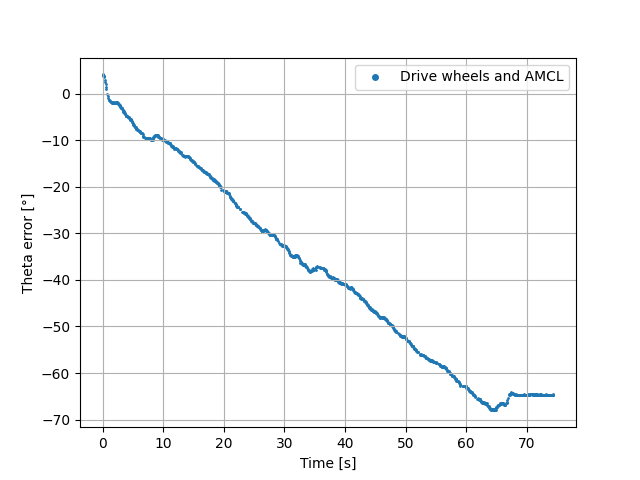

Supplement: Multimedia component 1 [file mmc1.zip › repository/orientation_errors/scenario3_dw_amcl.png]

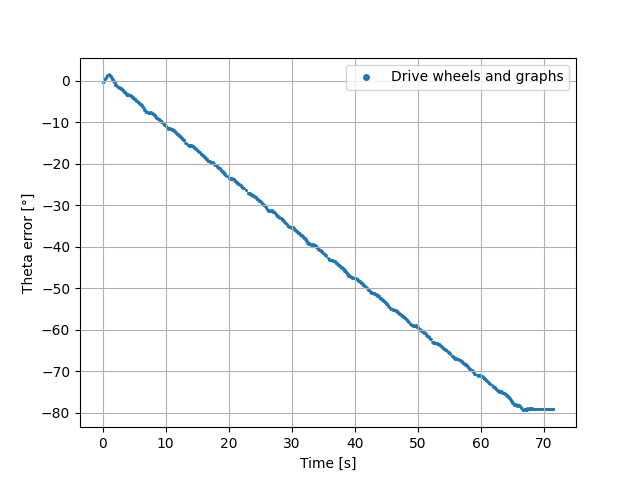

Supplement: Multimedia component 1 [file mmc1.zip › repository/orientation_errors/scenario3_dw_graphs.png]

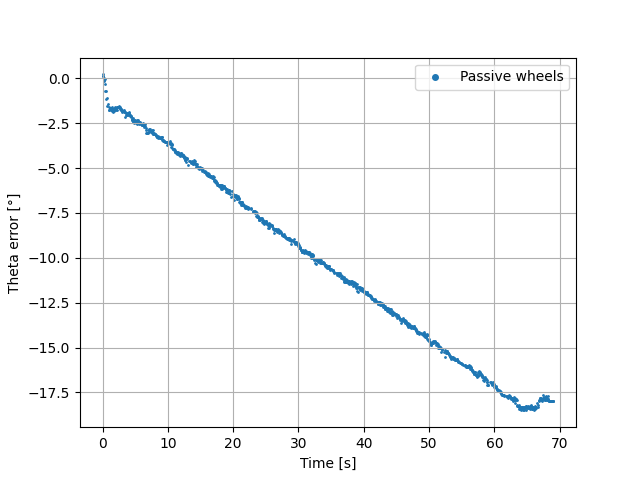

Supplement: Multimedia component 1 [file mmc1.zip › repository/orientation_errors/scenario3_pw.png]

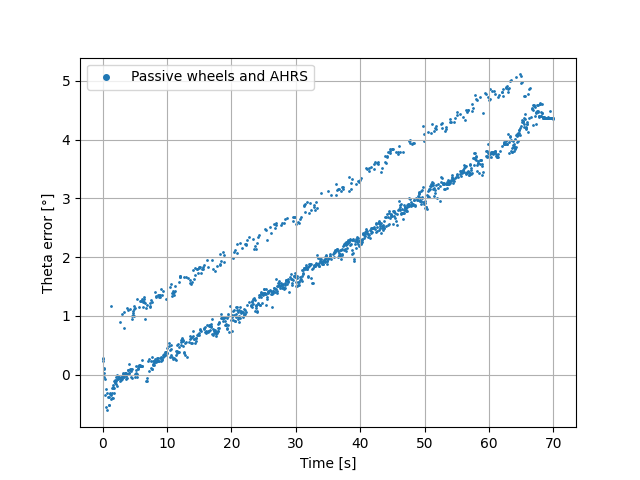

Supplement: Multimedia component 1 [file mmc1.zip › repository/orientation_errors/scenario3_pw_ahrs.png]

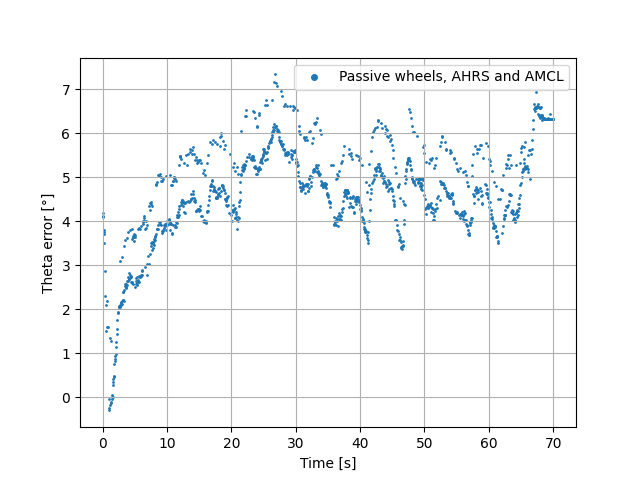

Supplement: Multimedia component 1 [file mmc1.zip › repository/orientation_errors/scenario3_pw_ahrs_amcl.png]

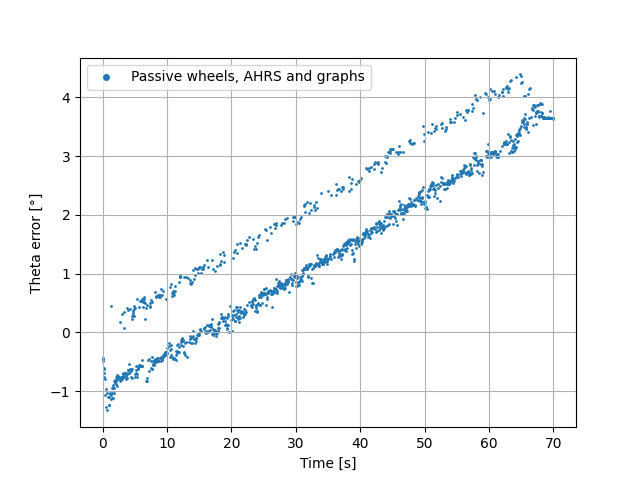

Supplement: Multimedia component 1 [file mmc1.zip › repository/orientation_errors/scenario3_pw_ahrs_graphs.png]

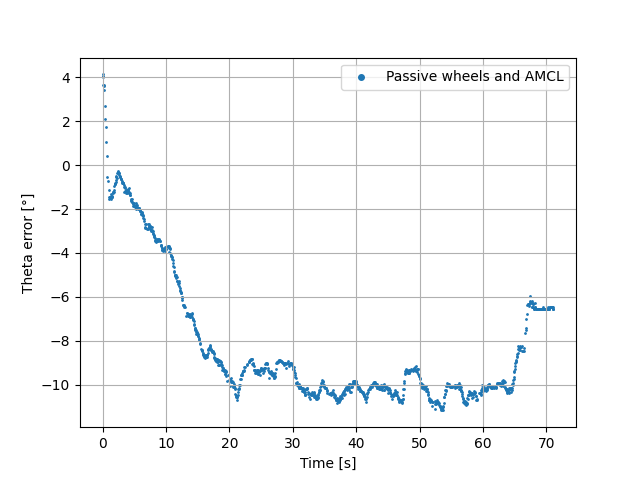

Supplement: Multimedia component 1 [file mmc1.zip › repository/orientation_errors/scenario3_pw_amcl.png]

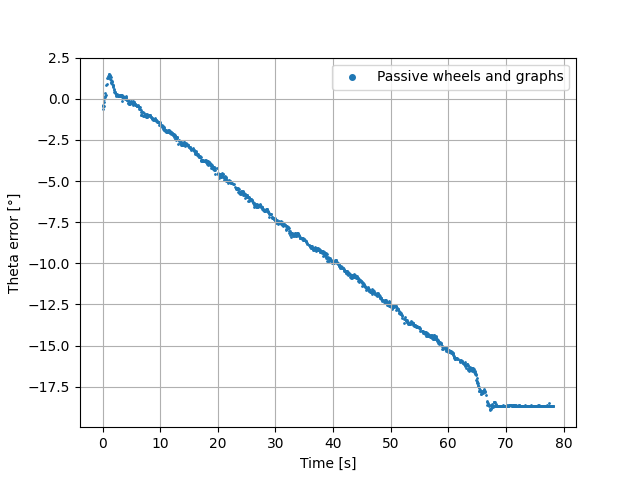

Supplement: Multimedia component 1 [file mmc1.zip › repository/orientation_errors/scenario3_pw_graphs.png]

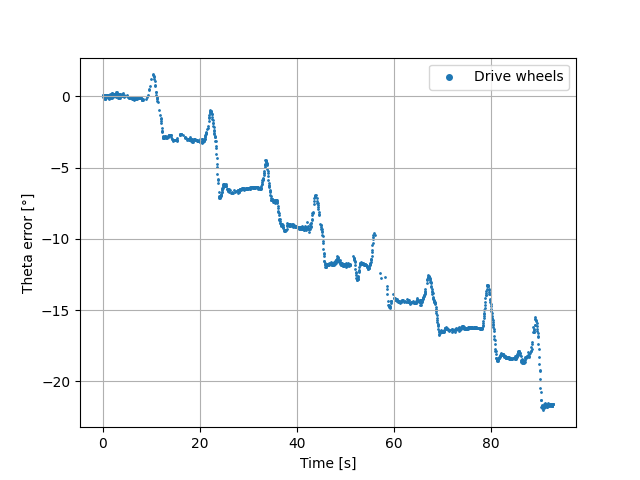

Supplement: Multimedia component 1 [file mmc1.zip › repository/orientation_errors/scenario4_dw.png]

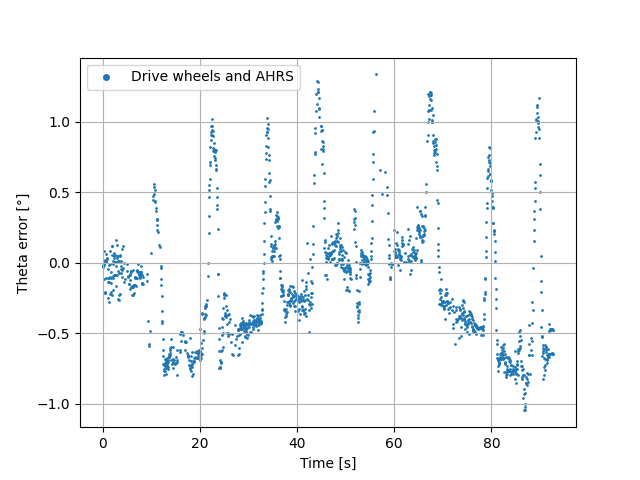

Supplement: Multimedia component 1 [file mmc1.zip › repository/orientation_errors/scenario4_dw_ahrs.png]

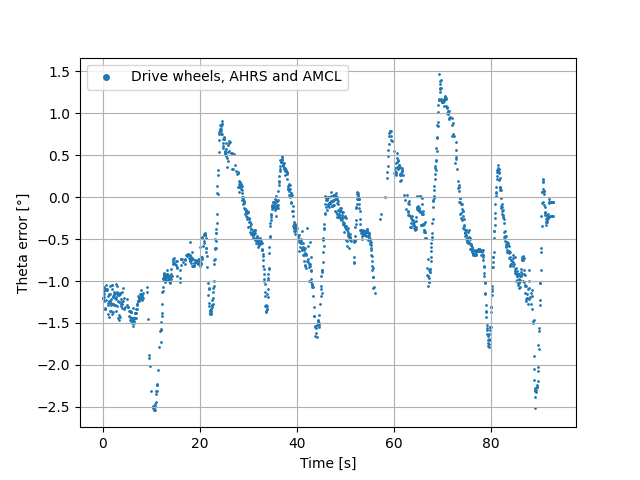

Supplement: Multimedia component 1 [file mmc1.zip › repository/orientation_errors/scenario4_dw_ahrs_amcl.png]

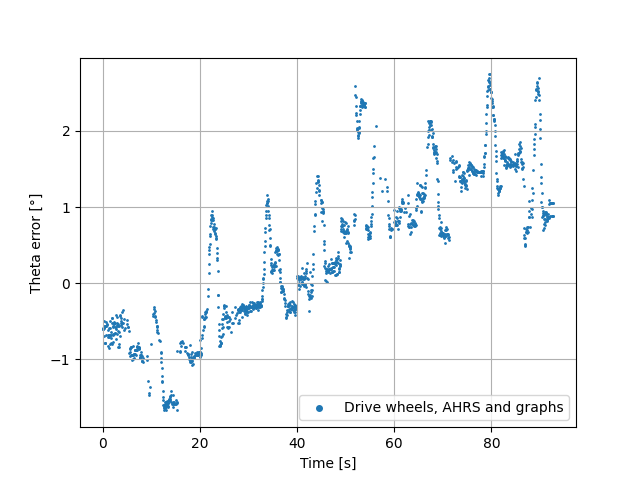

Supplement: Multimedia component 1 [file mmc1.zip › repository/orientation_errors/scenario4_dw_ahrs_graphs.png]

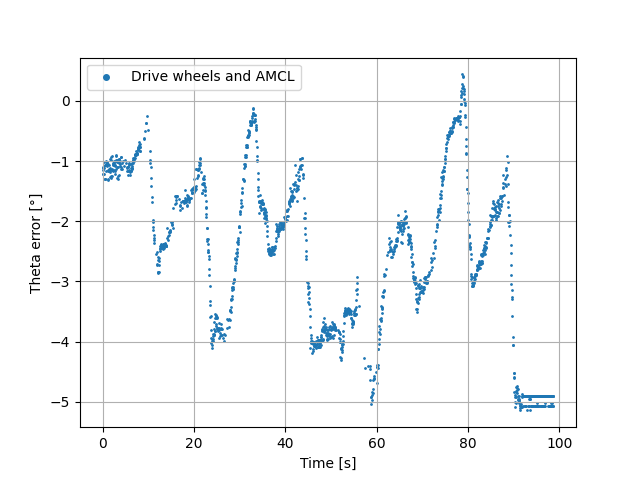

Supplement: Multimedia component 1 [file mmc1.zip › repository/orientation_errors/scenario4_dw_amcl.png]

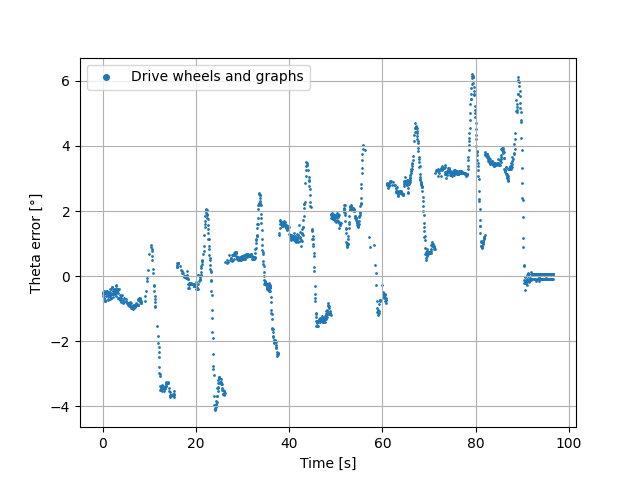

Supplement: Multimedia component 1 [file mmc1.zip › repository/orientation_errors/scenario4_dw_graphs.png]

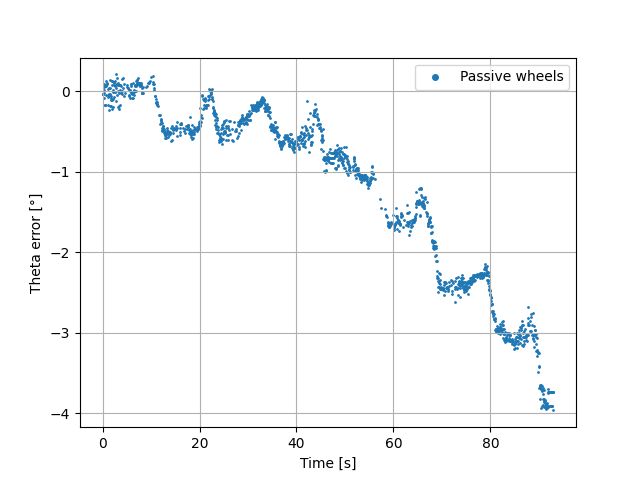

Supplement: Multimedia component 1 [file mmc1.zip › repository/orientation_errors/scenario4_pw.png]

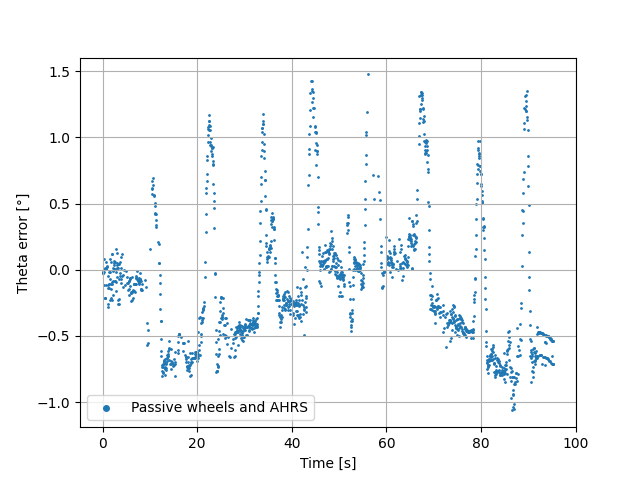

Supplement: Multimedia component 1 [file mmc1.zip › repository/orientation_errors/scenario4_pw_ahrs.png]

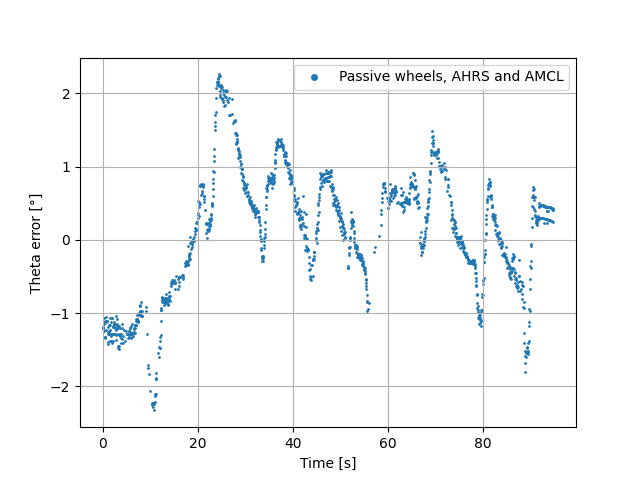

Supplement: Multimedia component 1 [file mmc1.zip › repository/orientation_errors/scenario4_pw_ahrs_amcl.png]

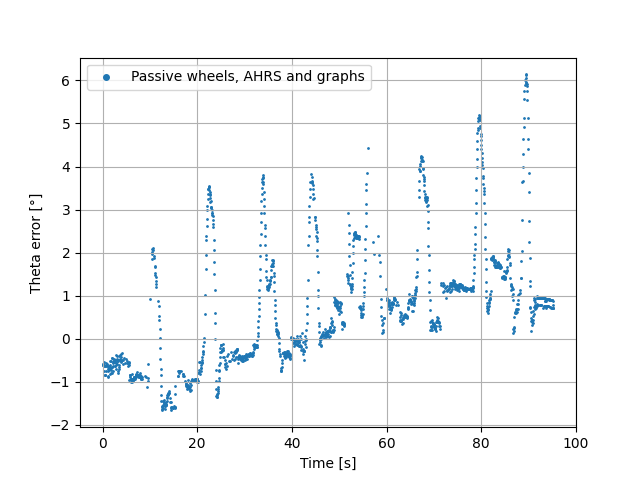

Supplement: Multimedia component 1 [file mmc1.zip › repository/orientation_errors/scenario4_pw_ahrs_graphs.png]

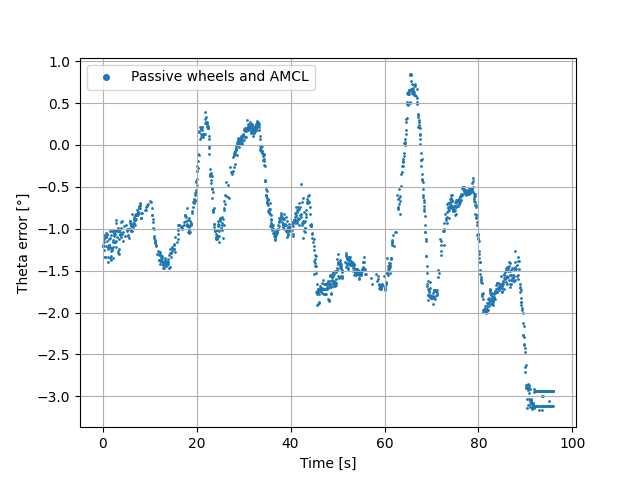

Supplement: Multimedia component 1 [file mmc1.zip › repository/orientation_errors/scenario4_pw_amcl.png]

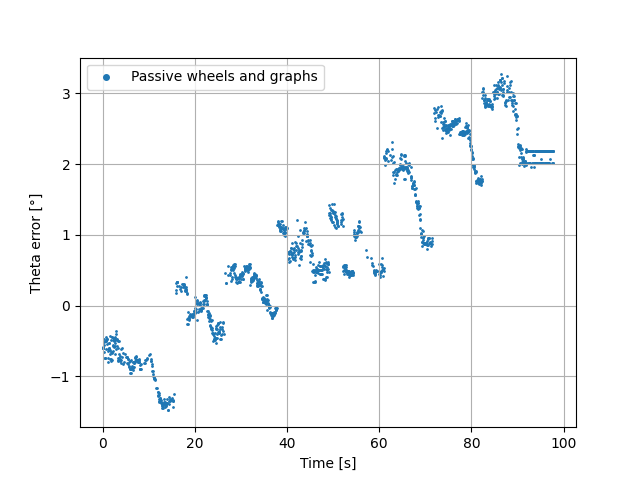

Supplement: Multimedia component 1 [file mmc1.zip › repository/orientation_errors/scenario4_pw_graphs.png]

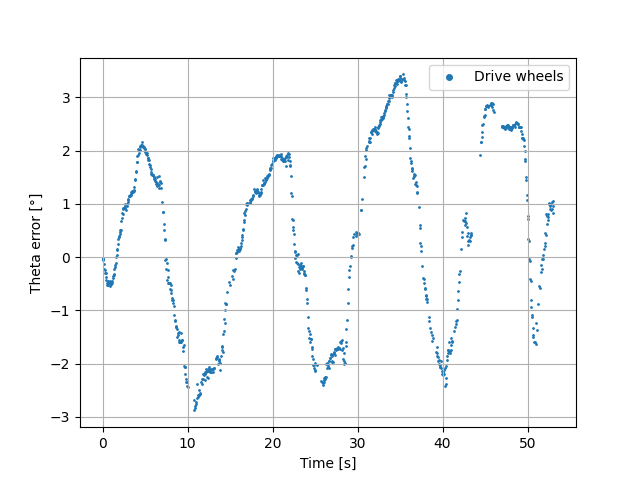

Supplement: Multimedia component 1 [file mmc1.zip › repository/orientation_errors/scenario5_dw.png]

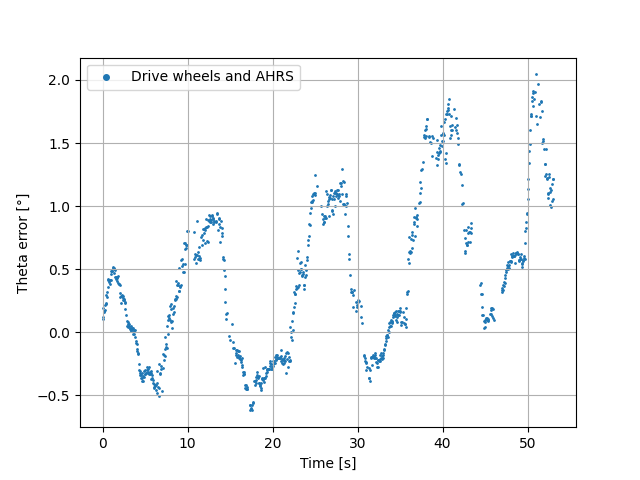

Supplement: Multimedia component 1 [file mmc1.zip › repository/orientation_errors/scenario5_dw_ahrs.png]

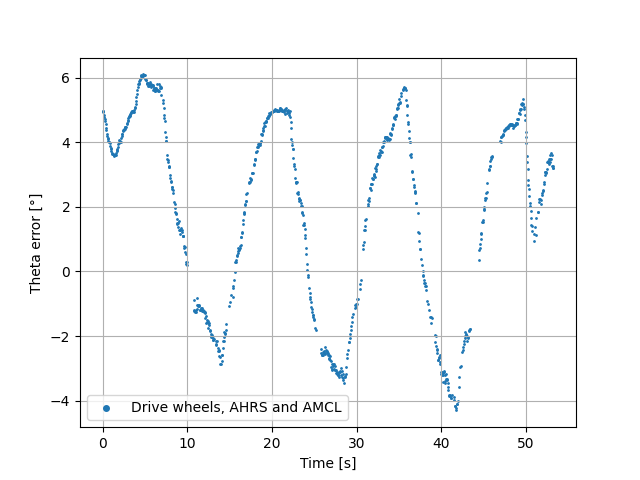

Supplement: Multimedia component 1 [file mmc1.zip › repository/orientation_errors/scenario5_dw_ahrs_amcl.png]

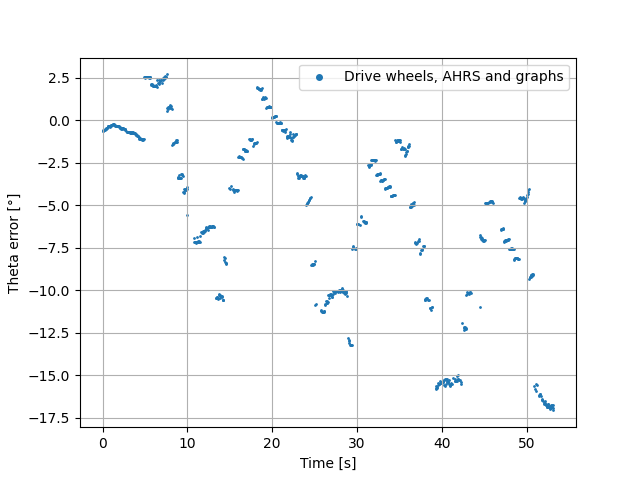

Supplement: Multimedia component 1 [file mmc1.zip › repository/orientation_errors/scenario5_dw_ahrs_graphs.png]

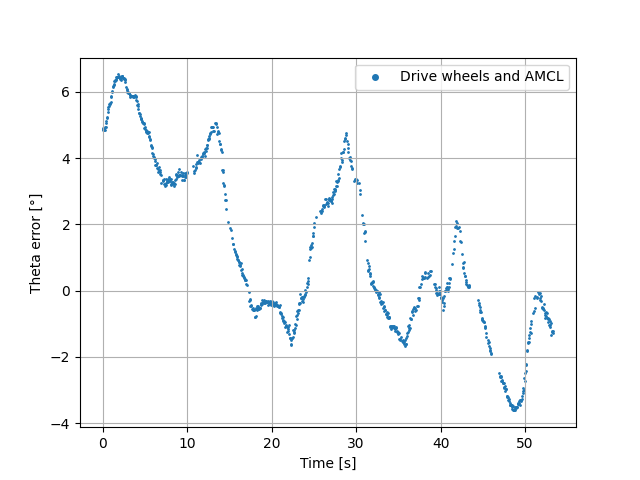

Supplement: Multimedia component 1 [file mmc1.zip › repository/orientation_errors/scenario5_dw_amcl.png]

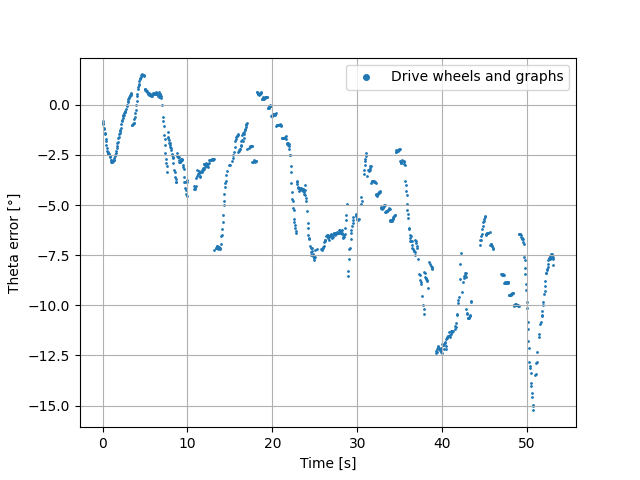

Supplement: Multimedia component 1 [file mmc1.zip › repository/orientation_errors/scenario5_dw_graphs.png]

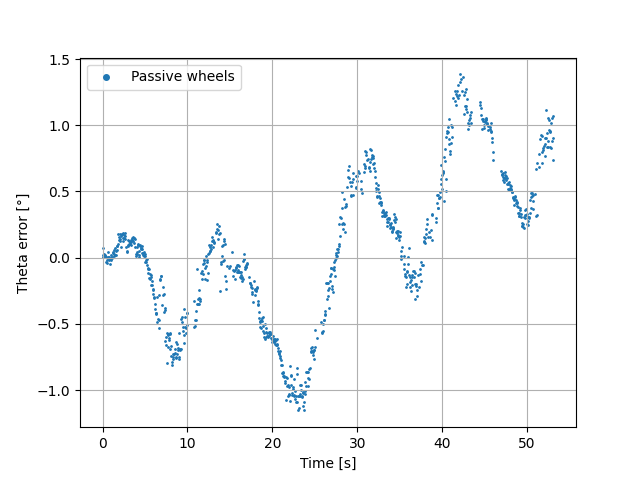

Supplement: Multimedia component 1 [file mmc1.zip › repository/orientation_errors/scenario5_pw.png]

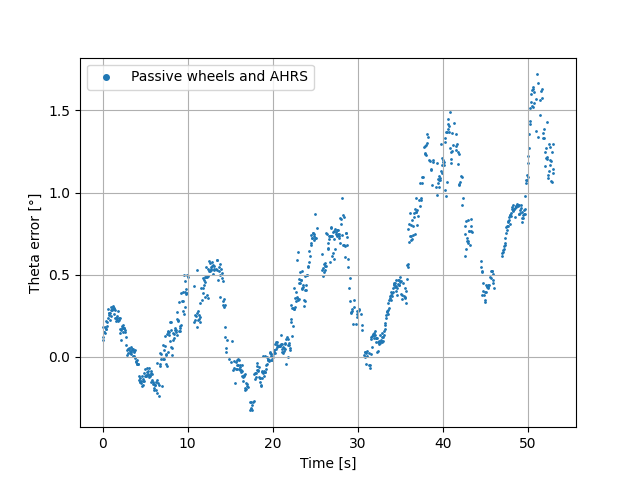

Supplement: Multimedia component 1 [file mmc1.zip › repository/orientation_errors/scenario5_pw_ahrs.png]

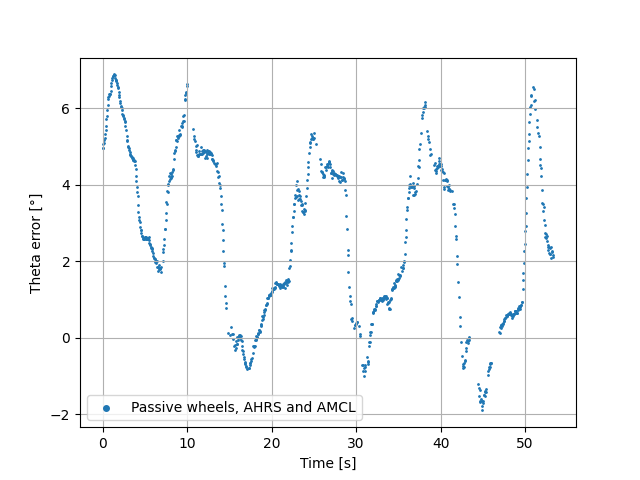

Supplement: Multimedia component 1 [file mmc1.zip › repository/orientation_errors/scenario5_pw_ahrs_amcl.png]

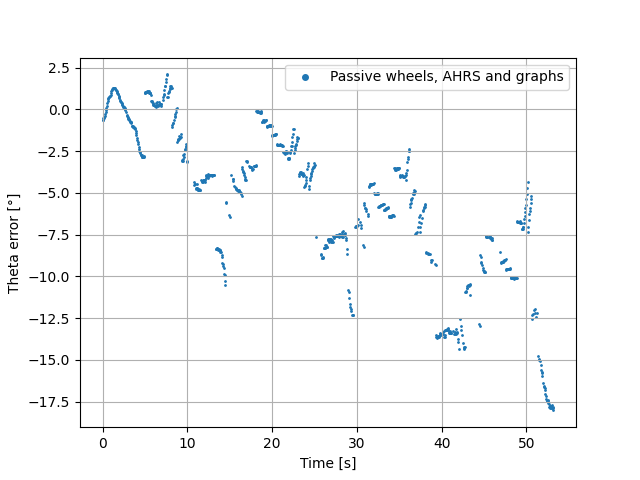

Supplement: Multimedia component 1 [file mmc1.zip › repository/orientation_errors/scenario5_pw_ahrs_graphs.png]

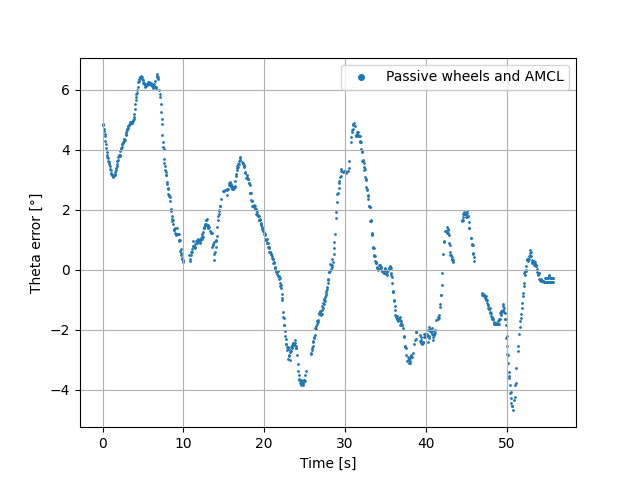

Supplement: Multimedia component 1 [file mmc1.zip › repository/orientation_errors/scenario5_pw_amcl.png]

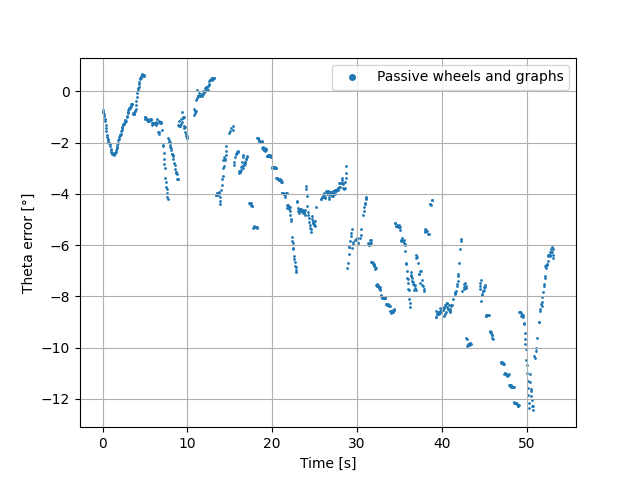

Supplement: Multimedia component 1 [file mmc1.zip › repository/orientation_errors/scenario5_pw_graphs.png]
